# Supplementary material for: Coordinated regulation of Rel expression by MAP3K4, CBP, and HDAC6 controls phenotypic switching
Source: Commun Biol. 2020 Aug 28;3:475. doi: 10.1038/s42003-020-01200-z (PMC7455715; doi:10.1038/s42003-020-01200-z)
Supplement: Supplementary file 1 — Supplementary Information [file 42003_2020_1200_MOESM1_ESM.pdf]

Coordinated regulation of Rel expression by MAP3K4, CBP, and HDAC6 controls phenotypic switching

Noha Ahmed Mohammed Shendy[1,2], Deepthi Raghu[1], Sujoy Roy[3], Charles Hamilton Perry[1], Adiba Safi[1], Miguel Ramos Branco[4], Ramin Homayouni[3], and Amy Noel Abell[1*]

Supplementary Figures

a

RPKM / Gene name	TS^{WT}	$TS^{WTCBPsh}$	TS^{KI4}	$TS^{KI4H6sh}$
Id2	143.89	85.62	63.38	116.87
Hoxa1	7.13	4.62	2.79	4.22
Runx1	2.13	0.63	0.40	0.80
Pou6f1	1.84	1.13	0.36	0.67
2610008E11Rik	1.68	1.02	1.22	1.86
Rel	1.67	1.22	0.30	0.69
Lhx6	1.60	1.02	1.02	1.65
Zfp672	1.06	0.60	0.56	0.86
Gli1	1.02	0.76	0.19	0.54
Ets1	1.02	0.55	0.36	1.02
Zfp810	0.97	0.67	0.41	0.66
Hivep2	0.89	0.66	0.21	0.37
Crebl2	0.88	0.48	0.27	0.69
Rfx3	0.74	0.33	0.42	0.77
Tlx1	0.66	0.31	0.28	0.50
Zfp273	0.47	0.31	0.31	0.68
Zfp784	0.30	0.19	0.14	0.21
Hmx2	0.19	0.12	0.05	0.10
Msc	0.12	0.03	0.07	0.15
Plag1	0.06	0.04	0.02	0.06
Zfp819	0.03	0.01	0	0.01
Rorc	0.01	0.01	0	0.01

b

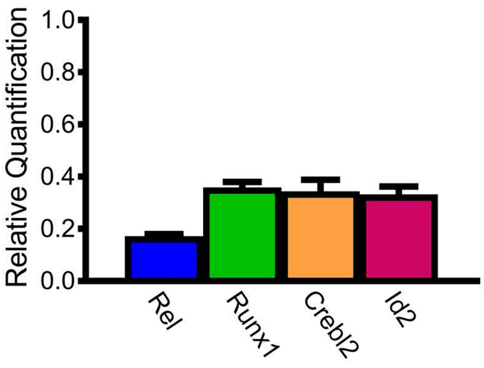

c

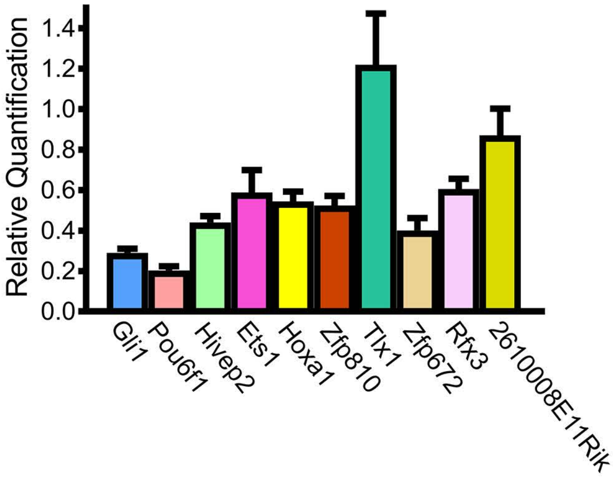

Supplementary Figure 1. Validation of expression of MAP3K4, CBP, HDAC6, and H2BK5Ac dependent transcription factors in TS cells. Related to Fig. 1. **a** Table shows reads per kilobase of transcript per million mapped reads (RPKM) from RNA-seq data for TFs co-regulated by MAP3K4, CBP, HDAC6, and H2BK5Ac. RNA-seq data were measured in TS^{WT} cells or TS^{KI4} cells expressing control shRNA, TS^{WT} cells expressing Crebbp shRNA ($TS^{WTCBPsh}$), or TS^{KI4} cells expressing Hdac6 shRNA ($TS^{KI4H6sh}$). **b**, **c** Validation of expression changes of TFs with RPKM value ≥ 0.5 in TS^{WT} cells using qPCR in TS^{WT} cells and TS^{KI4} cells. The data normalized to Actb (**b**) or Rps11 (**c**) are expressed as a fold-change relative to TS^{WT} cells and are the mean $\pm$ range of n=2 biologically independent experiments.

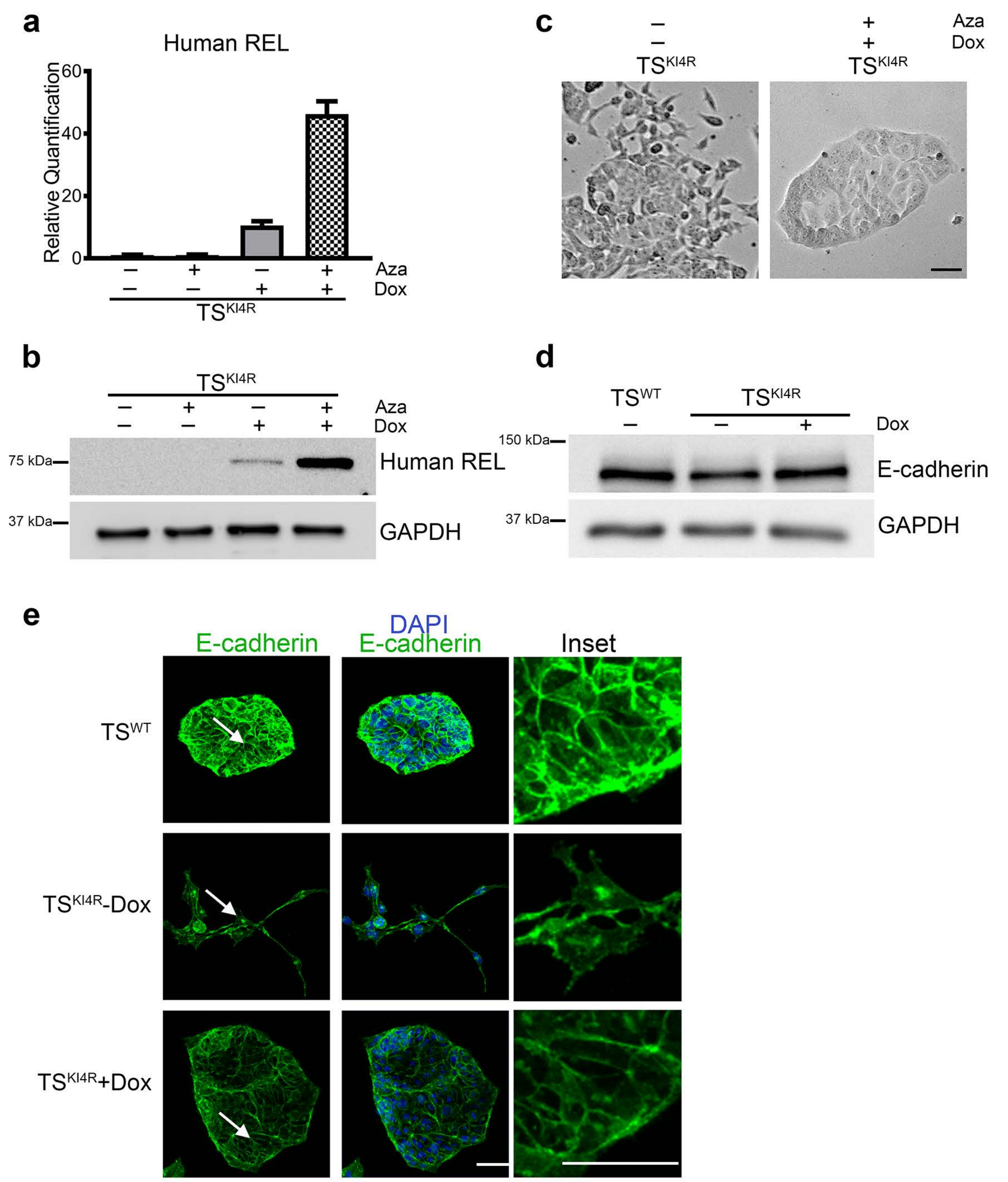

Supplementary Figure 2. Transient re-expression of REL in TS^{KI4} cells induces an epithelial morphology. Related to Fig. 3. **a** Human REL transcripts were measured in TS^{KI4} cells transduced with lentiviral constructs expressing Tet-on-human *REL*. Cells

were cultured for 96 hours as follows: in the absence of Doxycycline (Dox) and 5-aza-2'-deoxycytidine (Aza), in the presence of 1 μM Aza for 48 hours, in the presence of 2 μg/ml Dox for 96 hours, or in the presence of 1 μM Aza for 48 hours and 2 μg/ml Dox for 96 hours. Transcripts were measured using qPCR and human specific REL primers. qPCR data show the mean ± range of n=2 biologically independent experiments. **b** REL protein expression was measured using Western blotting and anti-human specific REL antibody. Blots of whole cell lysates derived from cells treated as in (**a**) are representative of n=3 biologically independent experiments. **c** Morphological changes induced by transient re-expression of REL in TS^{KI4} cells. Cells were either treated with DMSO or treated with 1 μM Aza for 48 hours and 2 μg/ml Dox for 96 hours. Representative phase microscopy images from n=3 biologically independent experiments are shown. Scale bar represents 400 μm. **d, e** Continuous growth of TS^{KI4R} cells in the absence or presence of Dox alters E-cadherin expression and localization. **(d)** Representative Western blots from n=3 biologically independent experiments are shown. **(e)** E-cadherin expression and localization in TS^{WT} cells, TS^{KI4R} cells transduced with lentiviral constructs expressing Tet-on-human *REL* cells cultured in the absence (-Dox) or presence (+Dox) of Doxycycline. Cells were stained with anti-E-cadherin antibody (green) and DAPI nuclear stain (blue). Arrows indicate the area of enlarged insets. Images are representative of n=3 biologically independent experiments. Scale bar represents 100 μm. Western blots show cropped images. Uncropped images are available in Supplementary Materials.

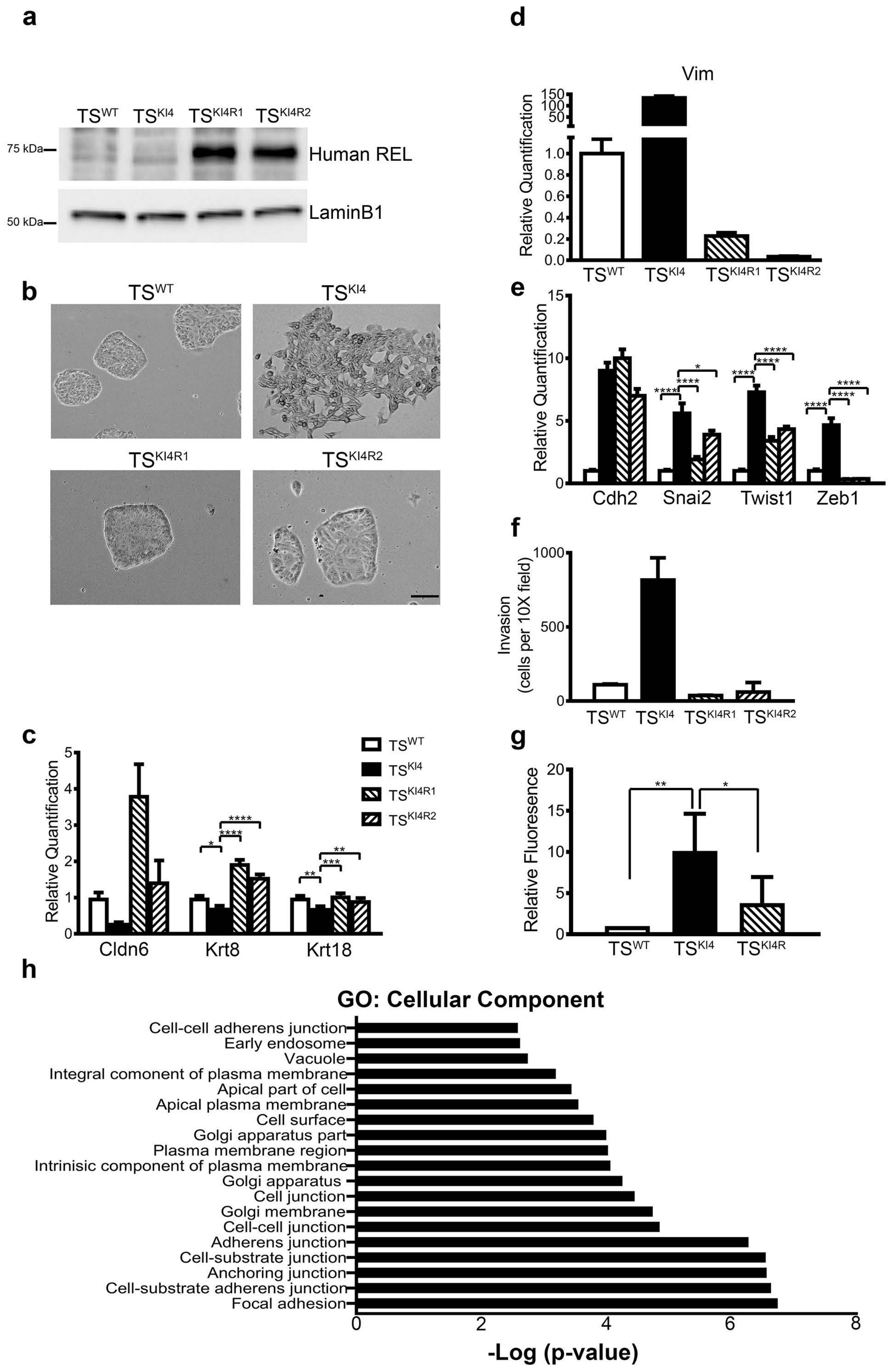

a
TSWT TSKI4 TSKI4R1 TSKI4R2
75 kDa
Human REL
50 kDa
LaminB1
b
TSWT
TSKI4
TSKI4R1
TSKI4R2
c
Relative Quantification
TSWT
TSKI4
TSKI4R1
TSKI4R2
Cldn6
Krt8
Krt18
d
Vim
Relative Quantification
TSWT TSKI4 TSKI4R1 TSKI4R2
e
Relative Quantification
Cdh2
Snai2
Twist1
Zeb1
f
Invasion (cells per 10X field)
TSWT TSKI4 TSKI4R1 TSKI4R2
g
Relative Fluoresence
TSWT TSKI4 TSKI4R
h
GO: Cellular Component
Cell-cell adherens junction
Early endosome
Vacuole
Integral comonent of plasma membrane
Apical part of cell
Apical plasma membrane
Cell surface
Golgi apparatus part
Plasma membrane region
Intrinisic component of plasma membrane
Golgi apparatus
Cell junction
Golgi membrane
Cell-cell junction
Adherens junction
Cell-substrate junction
Anchoring junction
Cell-substrate adherens junction
Focal adhesion
-Log (p-value)

Supplementary Figure 3. REL re-expression using a constitutive promoter restores the epithelial phenotype in TS^{KI4} cells. Related to Fig. 3. **a** Human REL expression analyzed in TS^{WT} cells, TS^{KI4} cells, and TS^{KI4} cells transduced with lentiviruses expressing human *REL* under a constitutive promoter (TS^{KI4R1} and TS^{KI4R2} cells) representing two independent clones. Western blotting analysis of nuclear lysates was performed using anti-human specific REL antibody. Blots are representative of n=3 biologically independent experiments. **b** TS^{KI4R1} and TS^{KI4R2} cells display an epithelial morphology. Representative phase microscopy images from n=3 biologically independent experiments are shown. Scale bar represents 400 μm. **c** Increased expression of epithelial markers at transcript level in TS^{KI4R1} and TS^{KI4R2} cells. Transcripts were measured using qPCR. The data normalized to Actb are expressed as a fold-change relative to TS^{WT} cells and are mean $\pm$ range of n=2 biologically independent experiments (Cldn6) or mean $\pm$ SEM of n=3 biologically independent experiments (Krt8 and Krt18). **d**, **e** REL re-expression reduces mesenchymal markers and EMT-inducing TFs. Transcripts were measured using qPCR. The data normalized to Actb are expressed as a fold-change relative to TS^{WT} cells and are mean $\pm$ range of n=2 biologically independent experiments (Vim and Cdh2) or mean $\pm$ SEM of n=3 biologically independent experiments (Snai2, Twist1, and Zeb1). **f** Expression of REL under a constitutive promoter decreases invasiveness in TS^{KI4R1} and TS^{KI4R2} cells relative to TS^{KI4} cells. Data show the mean $\pm$ range of n=2 biologically independent experiments performed in triplicate. **g** Barrier formation is increased upon re-expression of REL in TS^{KI4} cells as measured by fluorescent dye exclusion assays. Data show relative fluorescence compared to TS^{WT} cells and represent the mean $\pm$ SEM of n=4

biologically independent experiments. **h** Cellular component functional annotation of microarray data from TS^{KI4} cells and TS^{KI4} cells expressing human REL under a constitutive promoter (TS^{KI4R} cells). Genes whose expression is upregulated in TS^{KI4R} cells relative to TS^{KI4} cells were analyzed using a cut-off (log2(fold-change) $\geq$ 2 and FDR $<$ 0.05. *p-value < 0.05; **p-value < 0.01; ***p-value < 0.001, ****p-value < 0.0001; Student's t test. Western blots show cropped images. Uncropped images are available in Supplementary Materials.

a

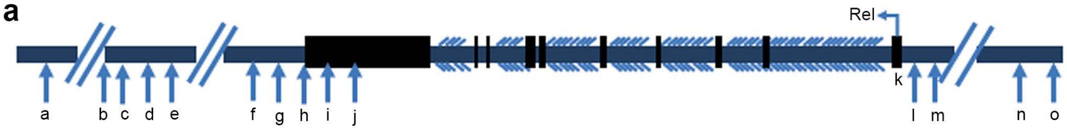

b

Region label	Region Description	Start site Chr11	End site Chr11	Promoter capture Hi-C reads number	Distance from Rel TSS (bp)
a	0610010F05Rik005 promoter	23630271	23635902	21	+140699
b	Pus10 promoter	23665391	23667090	96	+105579
c	Pus10 exon2 and intron2	23667213	23669040	37	+101930
d	Pus10 intron 4	23683782	23687879	19	+83091
e	Pus10 intron 4	23694210	23699820	25	+71150
f	Pus10 intron 15-17	23724472	23726153	39	+46498
g	Pus10 intron 17 and exon 18	23730289	23731740	41	+40681
h	Last exon of Rel and intergenic region between Rel and Pus10	23735153	23736974	33	+35817
i	Last exon of Rel	23737515	23741919	35	+33455
j	Last exon of Rel	23741920	23744690	38	+29050
k	Rel Promoter	23771650	23771728	NA	-700
l	Upstream Rel promoter	23780723	23787050	77	-9753
m	Upstream Rel promoter	23808076	23811028	33	-37106
n	Gm12061-001 promoter	23891217	23895635	76	-120247
o	A830031A19Rik-001 promoter	24068476	24075382	19	-297506

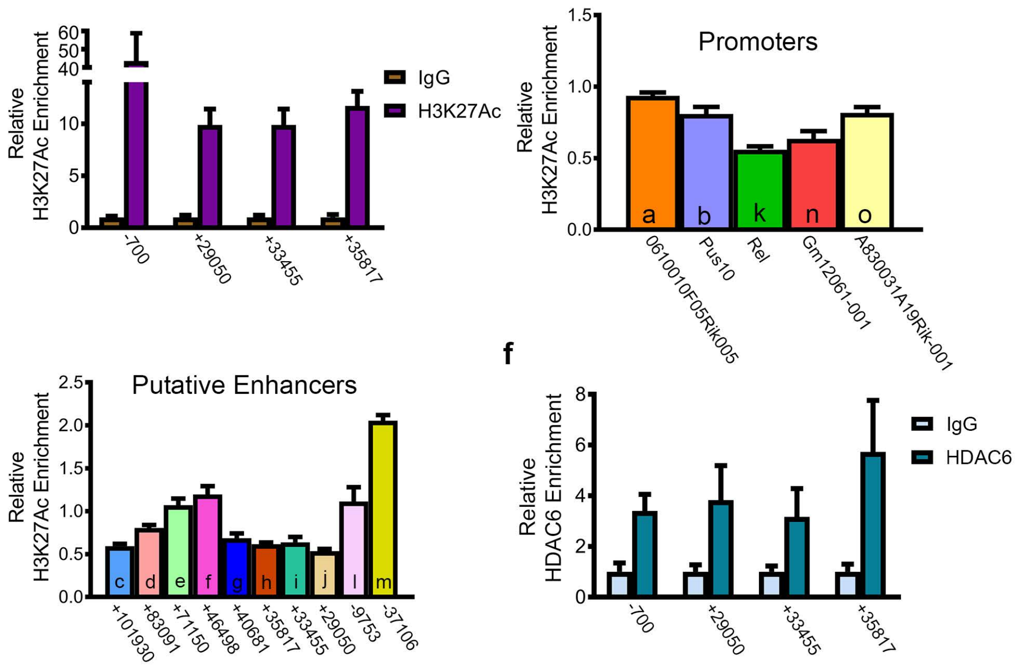

Supplementary Figure 4. Examination of predicted enhancer regions for *Rel* gene based on promoter capture Hi-C data using anti-H3K27Ac ChIP-PCR. Related to Fig. 5 and Fig. 6. **a** Schematic diagram of *Rel* locus. The arrows indicate locations of distal regions interacting with *Rel* promoter, labeled from (a-o) based on promoter capture Hi-C data in TS cells. **b** Table shows the region coordinates from the mm10 mouse genome, number of reads for the interactions called by GOTHiC from TS cell promoter capture Hi-C data, and distance from *Rel* transcription start site (TSS). **c** Enrichment of H3K27Ac on the *Rel* promoter and predicted enhancers in TS^{WT} cells compared to an isotype control IgG antibody was measured using anti-H3K27Ac and anti-IgG ChIP-PCR, respectively. Data shown are the mean ± range of n=2 biologically independent experiments. **d**, **e** Enrichment of H3K27Ac in TS^{KI4} cells relative to TS^{WT} cells was measured using anti-H3K27Ac ChIP-PCR. Fourteen sites were predicted by promoter capture Hi-C data to interact with the *Rel* promoter. These sites were examined by anti-H3K27Ac ChIP-PCR. Data are expressed as a fold change in TS^{KI4} cells relative to TS^{WT} cells. Sites were categorized as promoters (**d**) or putative enhancer regions (**e**). Data are the mean ± range of n=2 biologically independent experiments. **f** Enrichment of HDAC6 on the *Rel* promoter and putative enhancer regions in TS^{KI4} cells compared to an isotype control IgG antibody was measured using anti-HDAC6 and anti-IgG ChIP-PCR, respectively. Data shown are the mean ± range of n=2 biologically independent experiments.

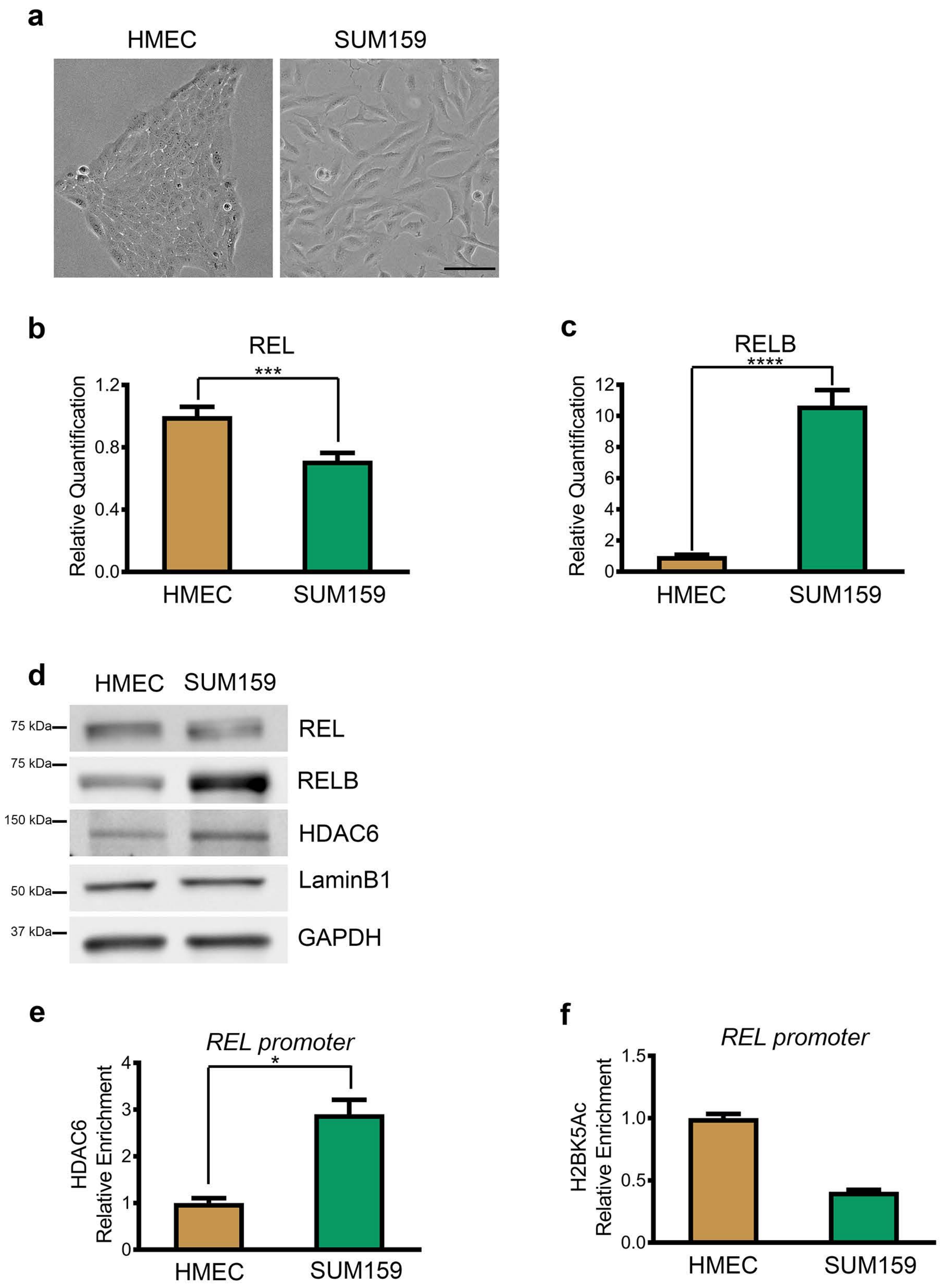

a
HMEC
SUM159
b
REL
***
Relative Quantification
1.2
0.8
0.4
0.0
HMEC
SUM159
c
RELB
****
Relative Quantification
12
10
8
6
4
2
0
HMEC
SUM159
d
HMEC
SUM159
75 kDa
REL
75 kDa
RELB
150 kDa
HDAC6
LaminB1
50 kDa
37 kDa
GAPDH
e
REL promoter
*
HDAC6
Relative Enrichment
4
3
2
1
0
HMEC
SUM159
f
REL promoter
H2BK5Ac
Relative Enrichment
1.5
1.0
0.5
0.0
HMEC
SUM159

Supplementary Figure 5. HDAC6 represses histone acetylation of the *REL* promoter in mesenchymal claudin-low breast cancer cells. Related to Fig. 6. **a** Phase microscopy images of epithelial human mammary epithelial cells (HMECs) and mesenchymal claudin-low SUM159 breast cancer cells. Black bar represents 400 μm. **b** REL transcript levels are reduced in SUM159s relative to HMECs. **c** Increased RELB expression at transcript level in SUM159s relative to HMECs. **b, c** Transcripts were measured using qPCR. The data normalized to GAPDH are expressed as a fold-change relative to HMECs and are the mean ± SEM of n=3 biologically independent experiments. **d** REL, RELB, and HDAC6 protein expression was measured using Western blotting. Blots of nuclear cell lysates are representative of n=3 biologically independent experiments. **e** Anti-HDAC6 ChIP-PCR shows increased HDAC6 enrichment on the *REL* promoter in SUM159s relative to HMECs. Data shown are the mean ± SEM of n=3 biologically independent experiments. **f** H2BK5Ac on the *REL* promoter is reduced in SUM159s relative to HMECs as measured by anti-H2BK5Ac ChIP-PCR. Data shown are the mean ± range of n=2 biologically independent experiments. *p-value < 0.05; ***p-value < 0.001, ****p-value < 0.0001; Student's t test.

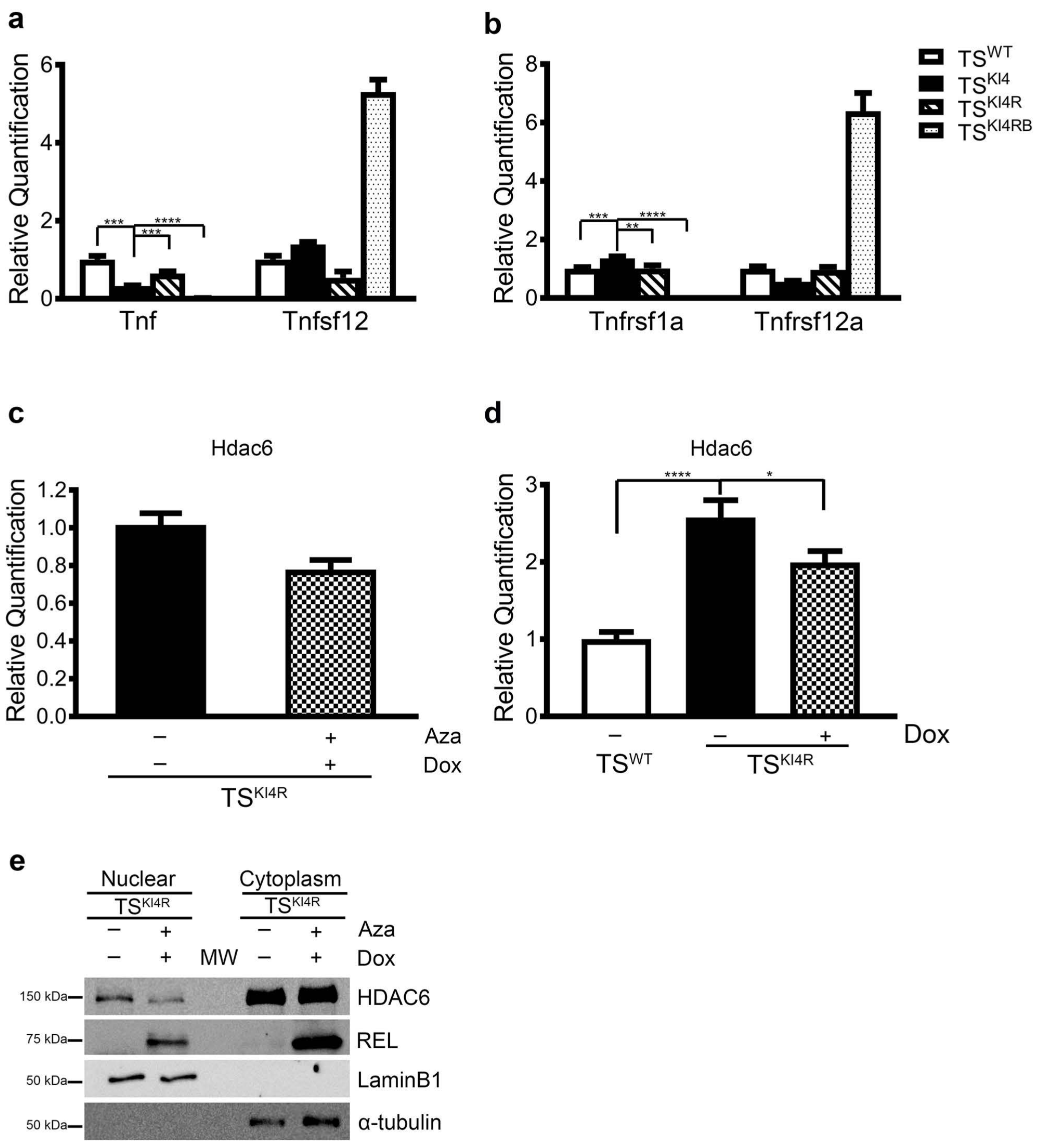

Supplementary Figure 6. REL expression in TS^{KI4R} cells does not induce the expression of inflammatory cytokines or their receptors, and transient REL expression is sufficient to reduce HDAC6 expression and nuclear localization. Related to Fig. 7. **a** Transcript expression of inflammatory cytokines, Tnf and Tnfsf12, in TS^{KI4} cells constitutively expressing REL (TS^{KI4R}) and TS^{KI4} cells constitutively expressing RELB (TS^{KI4RB}). **b** Transcript expression of cytokine receptors, Tnfrsf1a and Tnfrsf12a, in

TS^{KI4R} and TS^{KI4RB} cells. **a**, **b** Transcripts were measured using qPCR. The data normalized to Rps11 are expressed as a fold-change relative to TS^{WT} cells and mean ± SEM of n=3 biologically independent experiments (Tnf and Tnfrsf1a) or are mean ± range of n=2 biologically independent experiments (Tnfsf12 and Tnfrsf12a). **c** Transient induction of human REL expression decreases Hdac6 transcript levels. Hdac6 transcripts were measured in TS^{KI4} cells transduced with lentiviral constructs expressing Tet-on-human *REL*. Cells were either treated with DMSO or treated with 1 µM Aza for 48 hours and 2 µg/ml Dox for 96 hours. Transcripts were measured using qPCR. The data normalized to Rps11 are expressed as a fold-change relative to TS^{KI4R} cells treated with DMSO and are the mean ± range of n=2 biologically independent experiments.
d Hdac6 expression in TS^{KI4} cells expressing an inducible *Rel* construct and treated continuously with Dox. qPCR show the mean ± SEM of n=3 biologically independent experiments. **e** Transient human REL expression in TS^{KI4R} cells decreases nuclear HDAC6 protein levels. Protein expression was measured in cytoplasmic and nuclear extracts. Data show representative images from n=2 biologically independent experiments. *p-value < 0.05; **p-value < 0.01; ***p-value < 0.001, ****p-value < 0.0001; Student's t test.

Supplementary Figure 7. Uncropped Western blots.

Fig. 2b

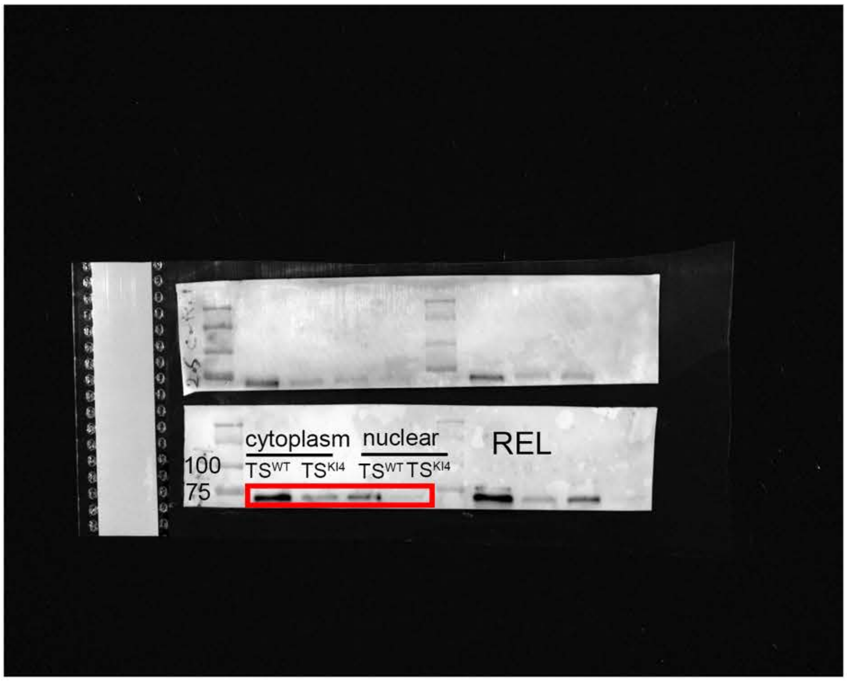

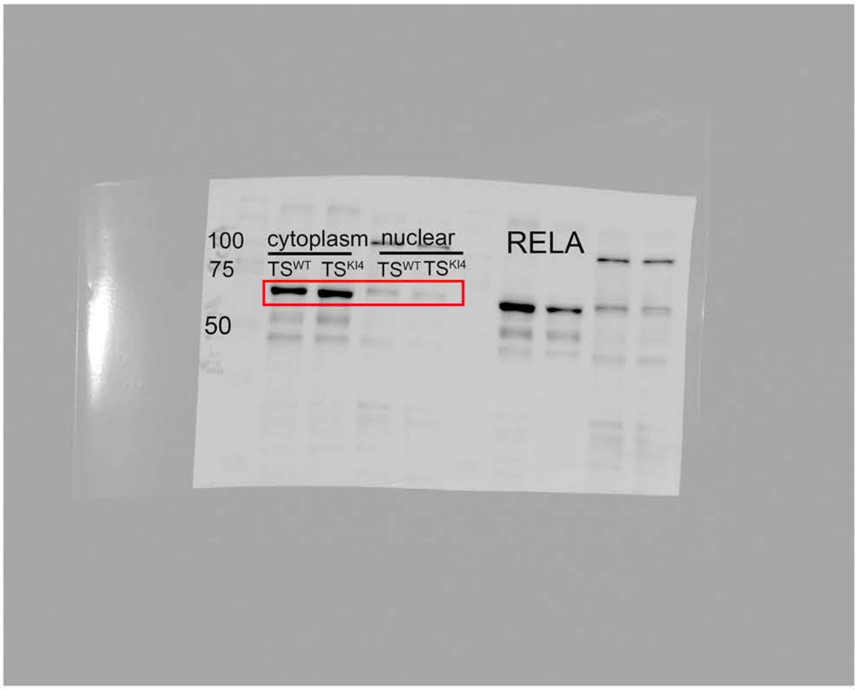

Fig. 2b

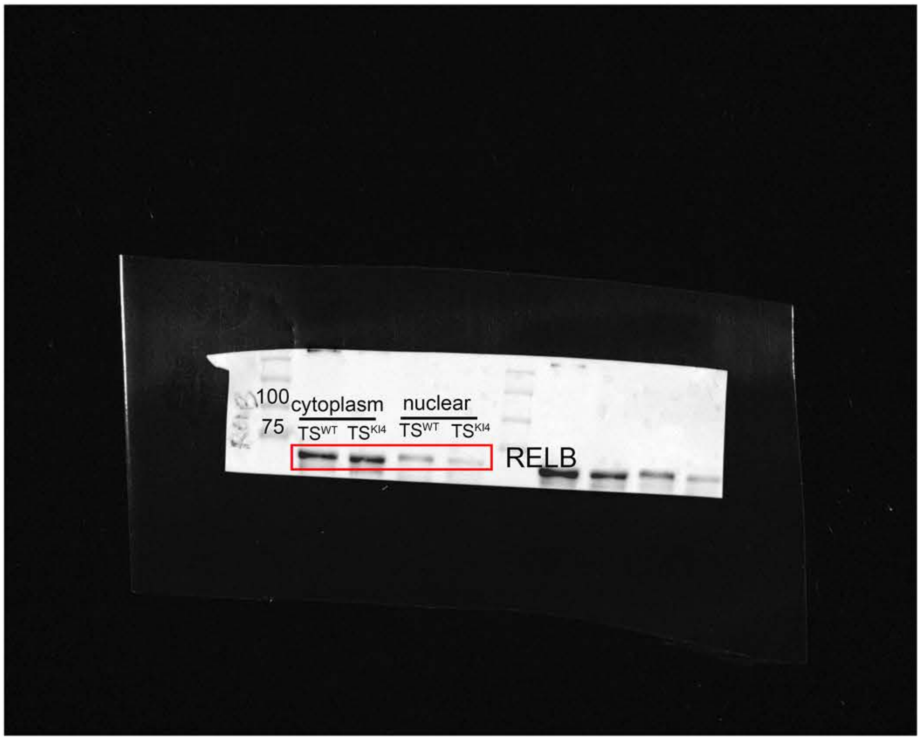

100
75
cytoplasm
nuclear
TSWT TSKI4 TSWT TSKI4
RELB

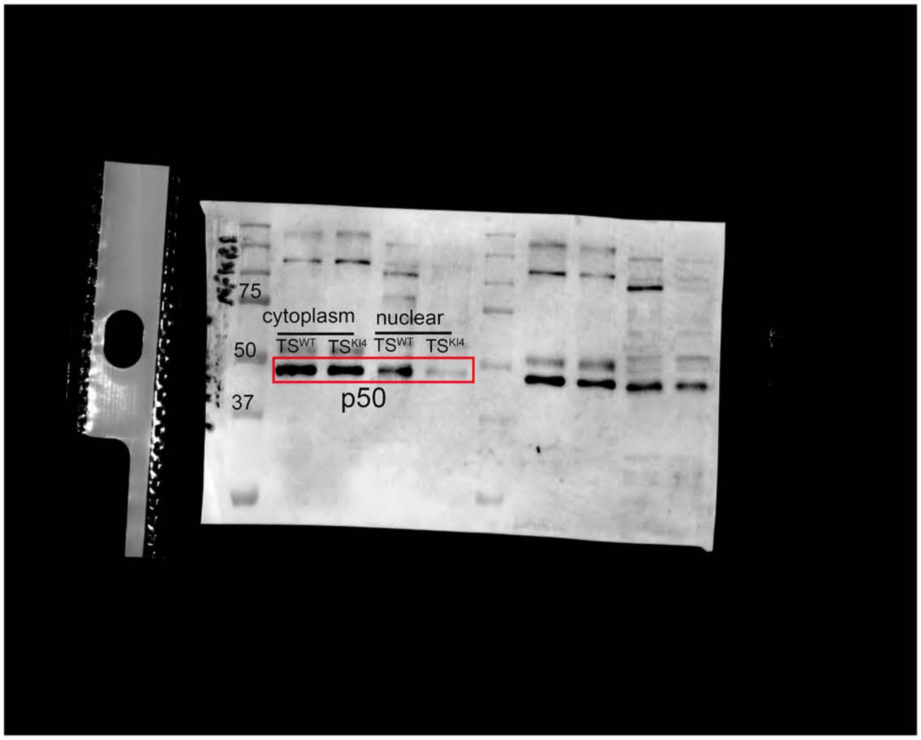

75
50
37
cytoplasm
nuclear
TSWT TSKI4 TSWT TSKI4
p50

Fig. 2b

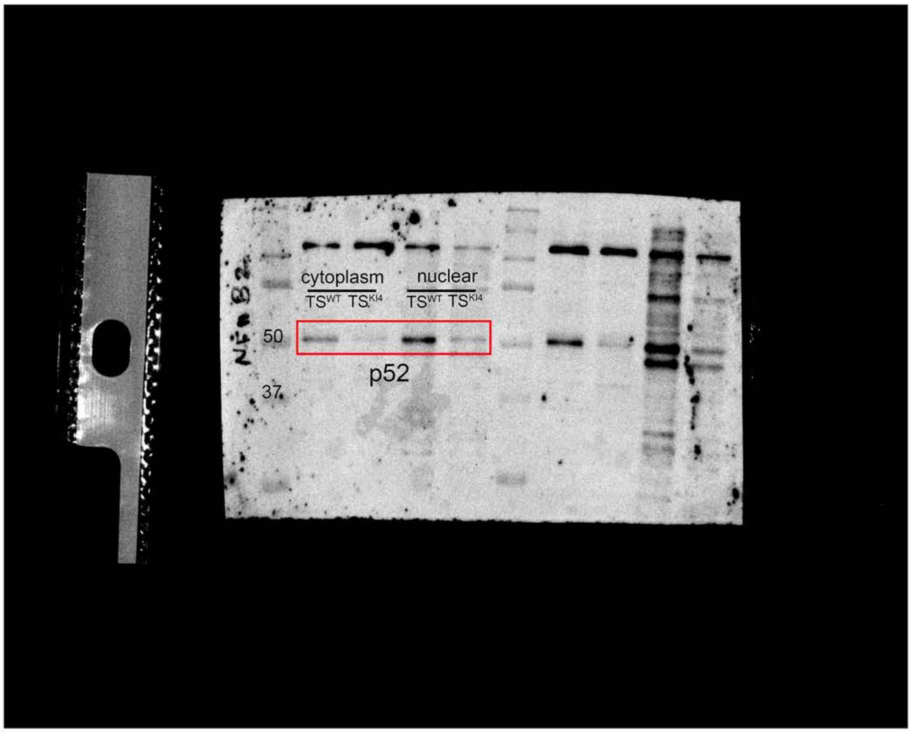

cytoplasm
TSWT TSKI4
nuclear
TSWT TSKI4
50
37
p52

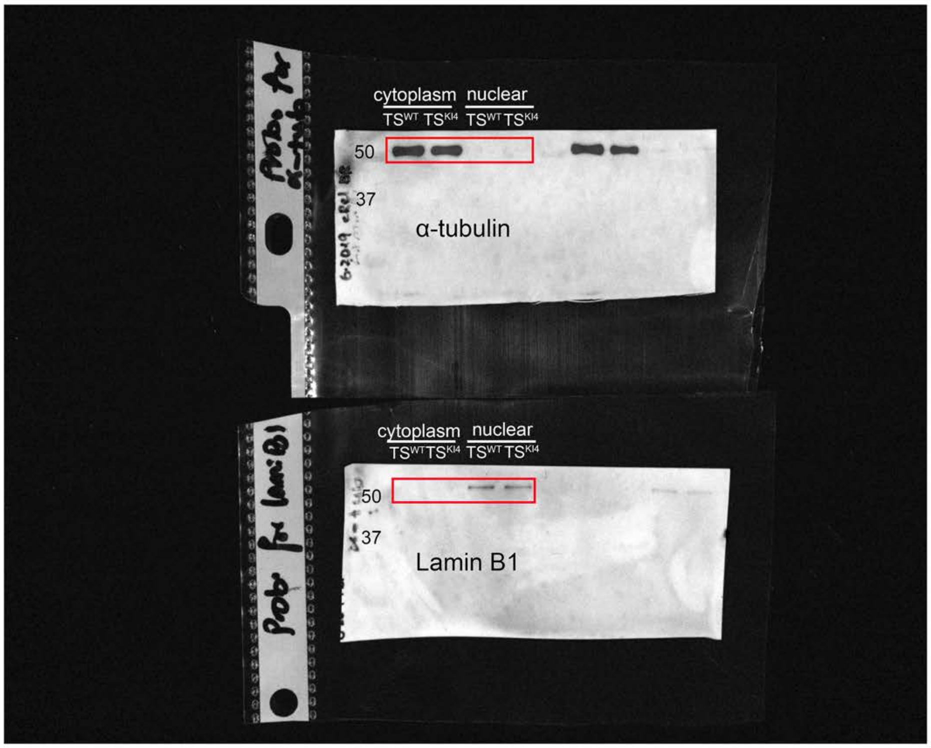

cytoplasm
TSWT TSKI4
nuclear
TSWT TSKI4
50
37
α-tubulin
cytoplasm
TSWT TSKI4
nuclear
TSWT TSKI4
50
37
Lamin B1

Fig. 2b

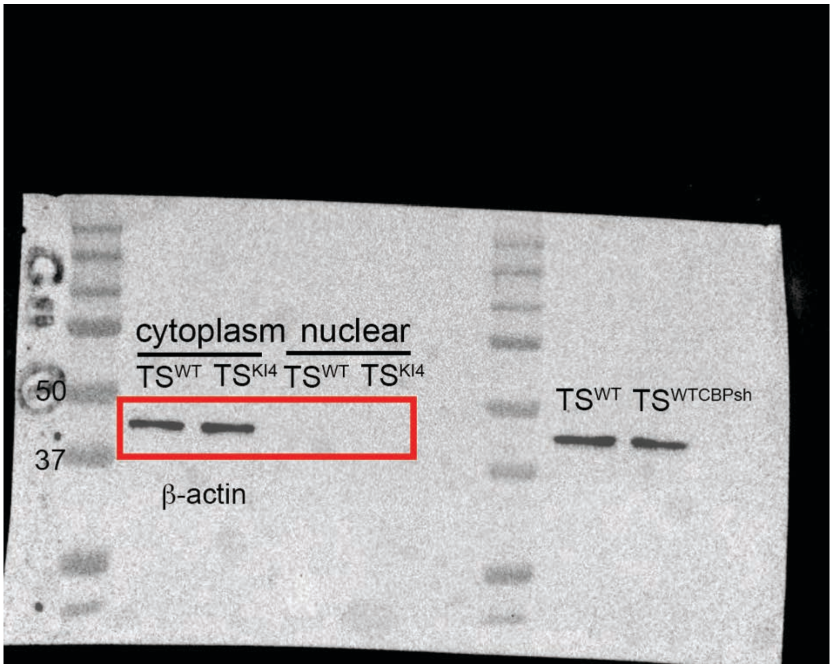
cytoplasm
nuclear
TSWT TSKI4 TSWT TSKI4
50
37
β-actin
TSWT TSWTCBPsh

Fig. 3b

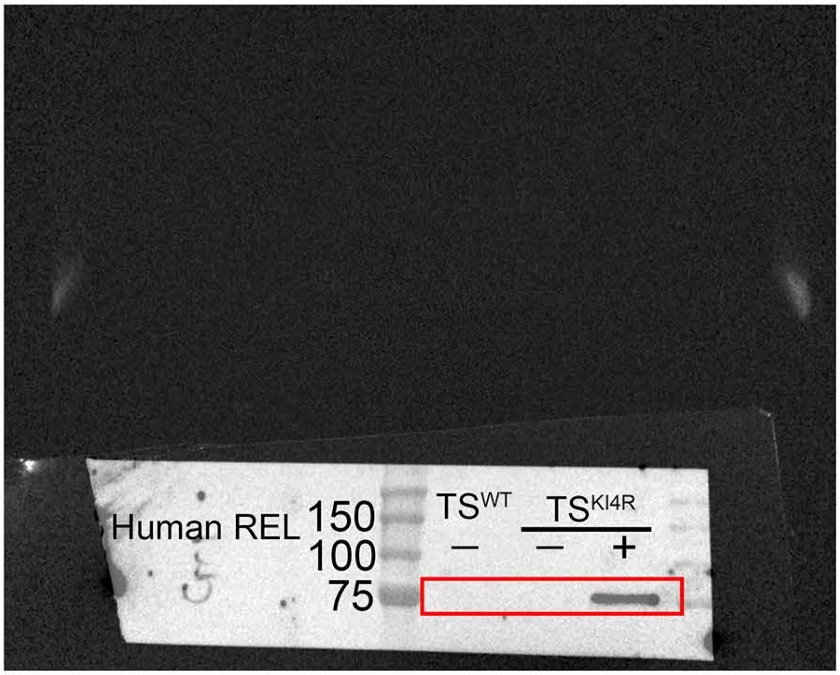

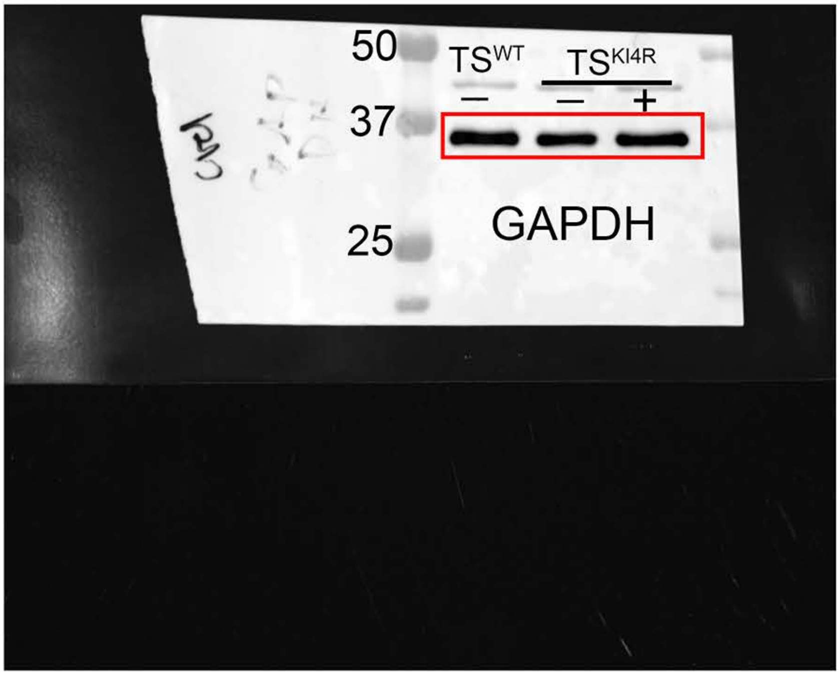

Fig. 4a

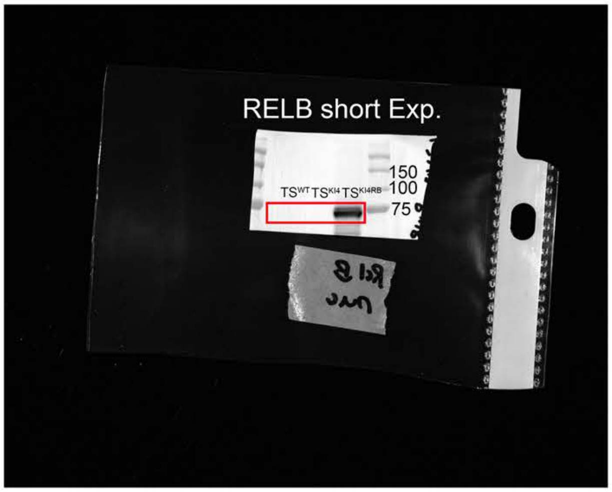
RELB short Exp.
TSWT TSKI4 TSKI4RB
150
100
75

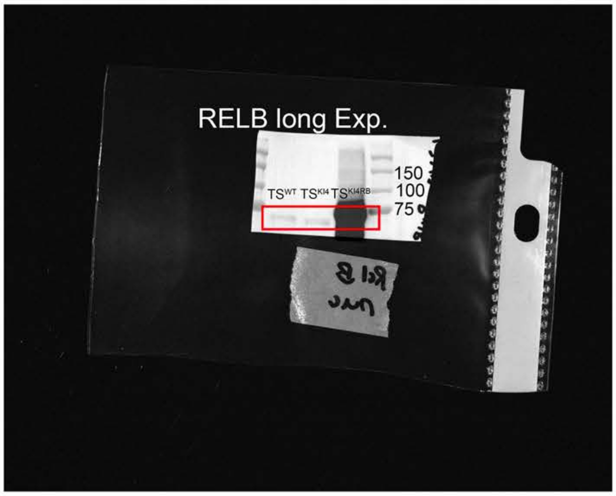
RELB long Exp.
TSWT TSKI4 TSKI4RB
150
100
75

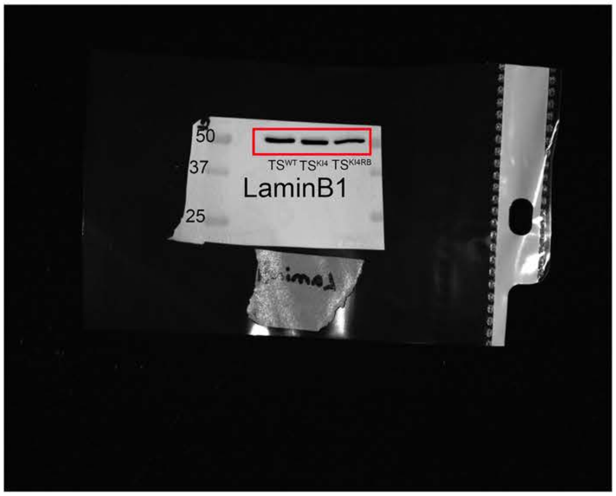
50
37
25
TSWT TSKI4 TSKI4RB
LaminB1

Fig. 5d

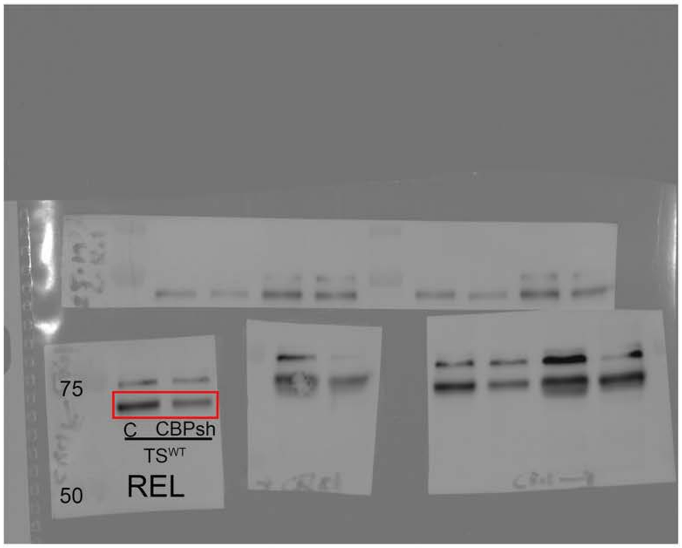

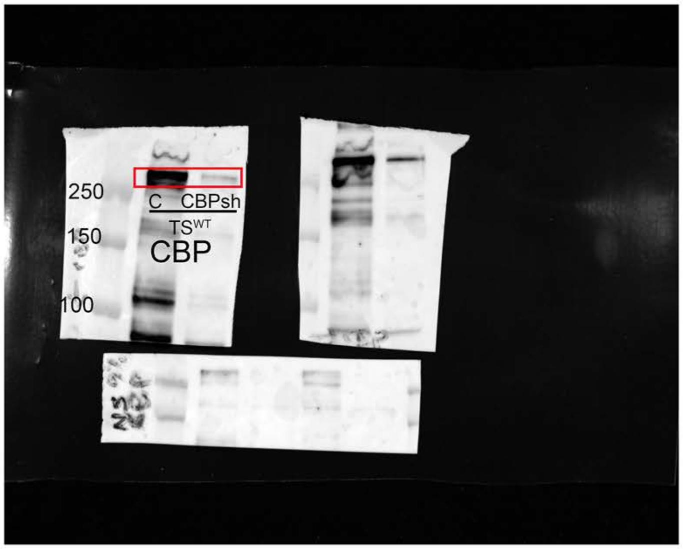

Fig. 5d

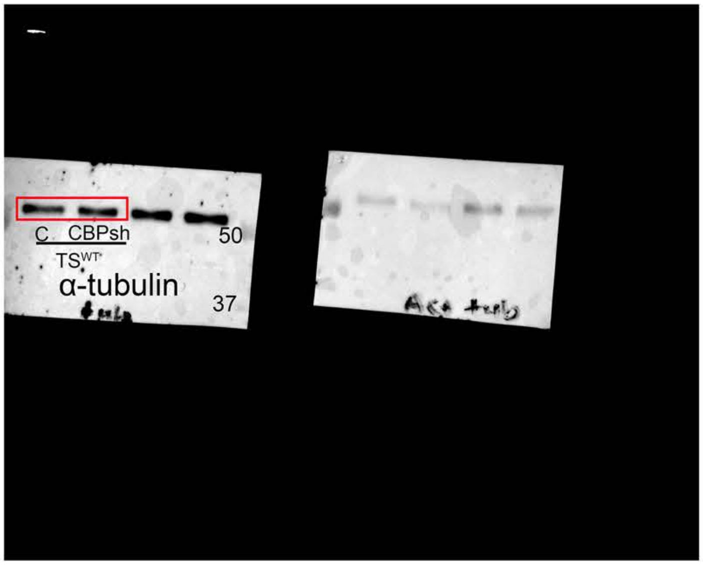
C CBPsh
TSWT
α-tubulin
50
37

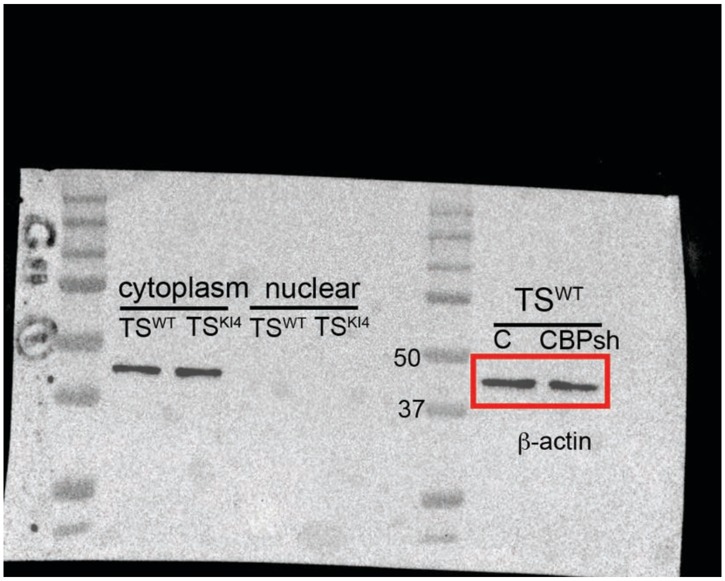
cytoplasm nuclear
TSWT TSKI4 TSWT TSKI4
TSWT
C CBPsh
50
37
β-actin

Fig. 5d

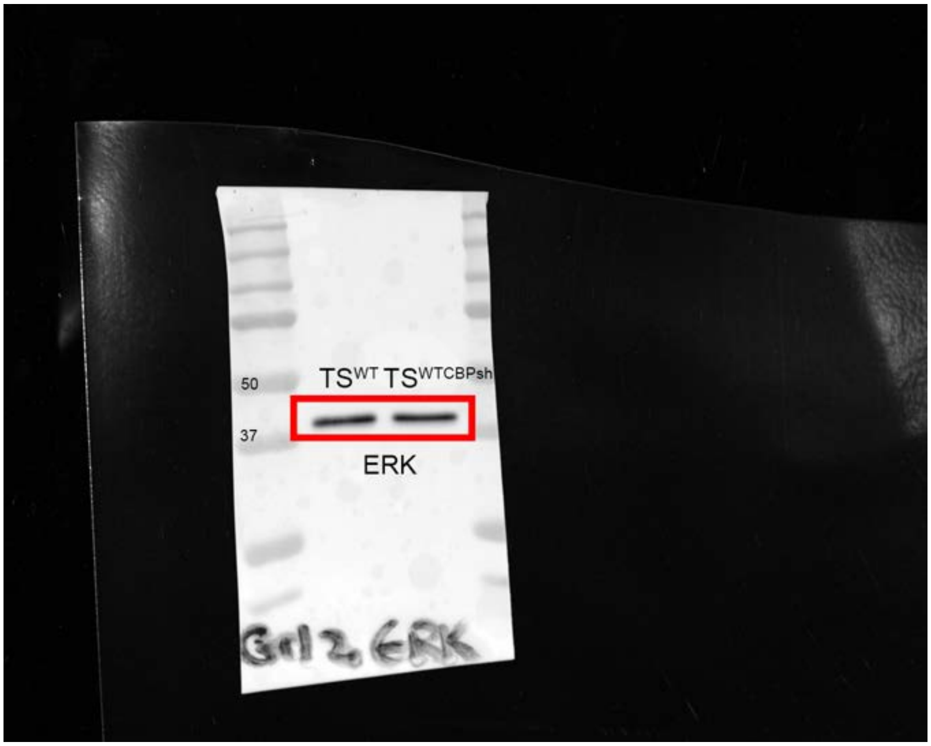

Fig. 6c

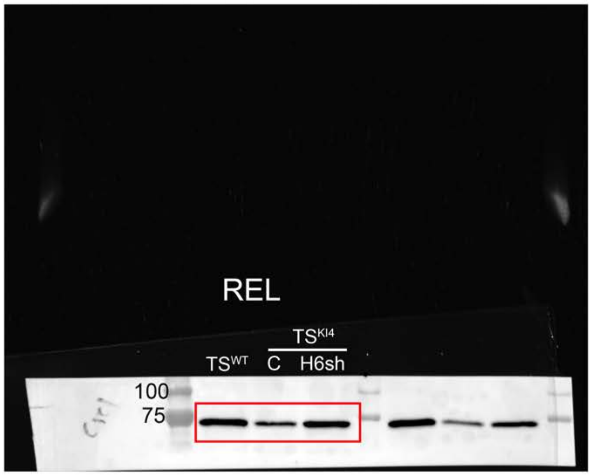
REL
TSKI4
TSWT C H6sh
100
75

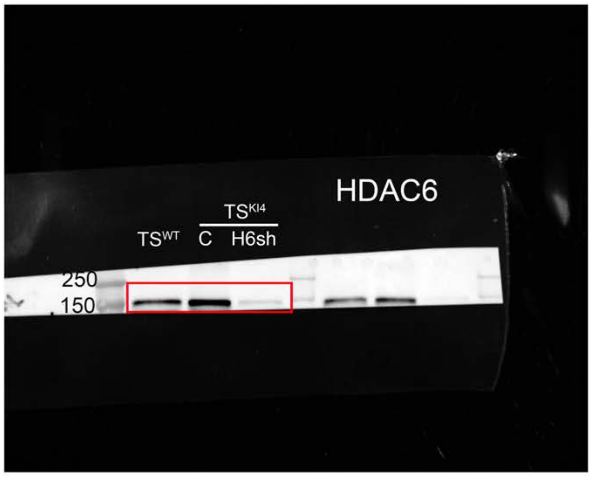
HDAC6
TSKI4
TSWT C H6sh
250
150

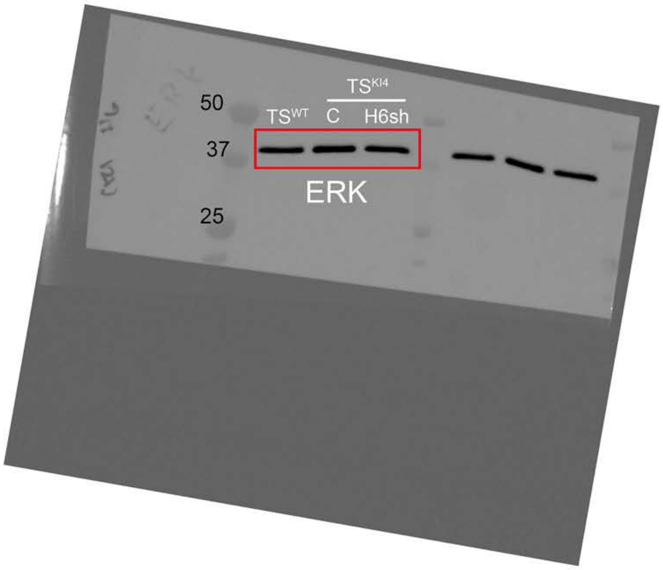
TSKI4
TSWT C H6sh
50
37
ERK
25

Fig. 6c

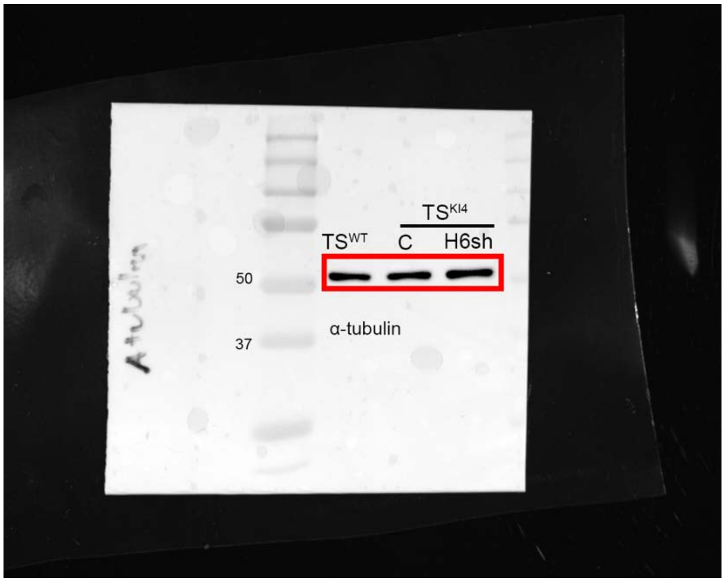
TS^KI4
TS^WT
C
H6sh
50
37
α-tubulin

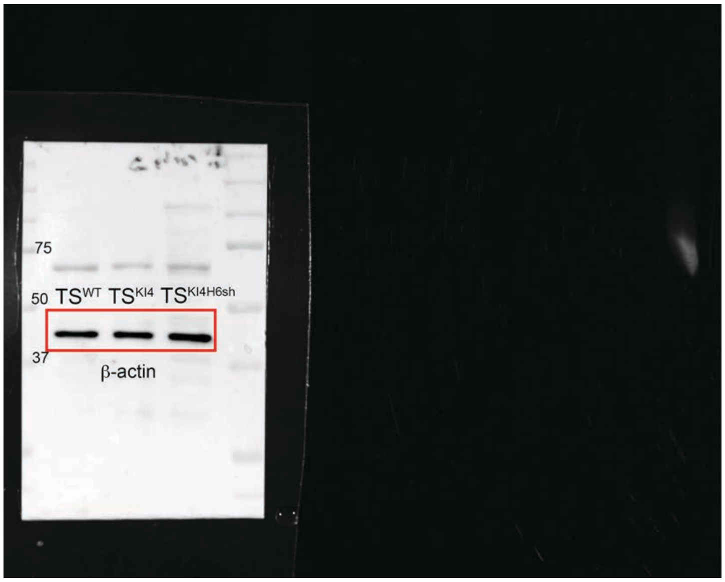
75
50
TS^WT
TS^KI4
TS^KI4H6sh
37
β-actin

Fig. 6f

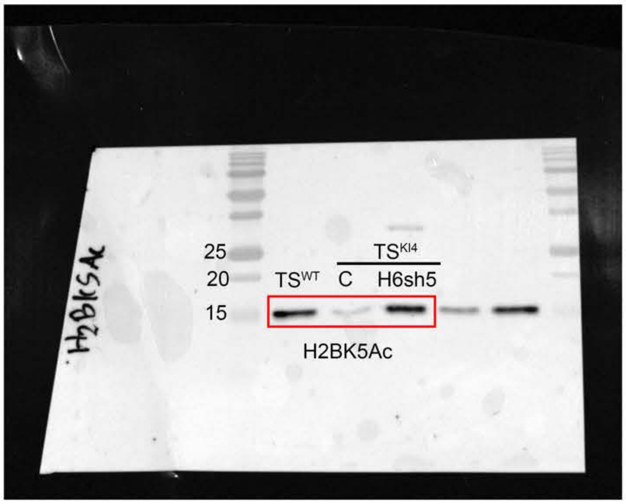
25
20
15
TS^KI4
TS^WT
C
H6sh5
H2BK5Ac

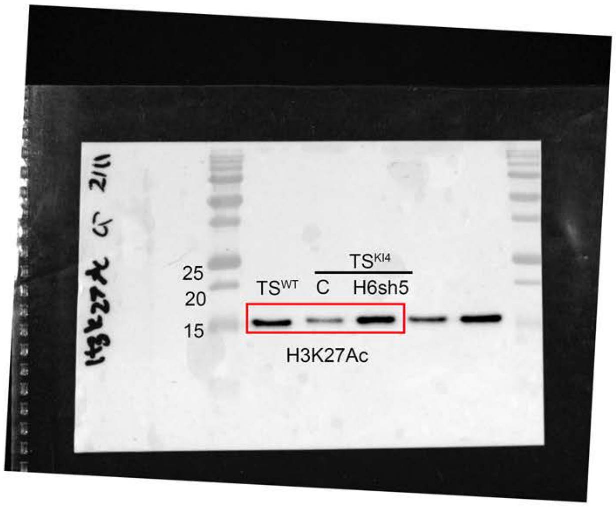
25
20
15
TS^KI4
TS^WT
C
H6sh5
H3K27Ac

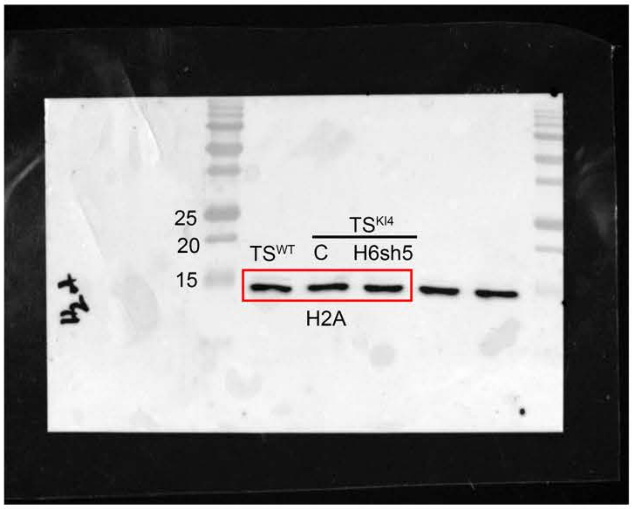
25
20
15
TS^KI4
TS^WT
C
H6sh5
H2A

Fig. 7g

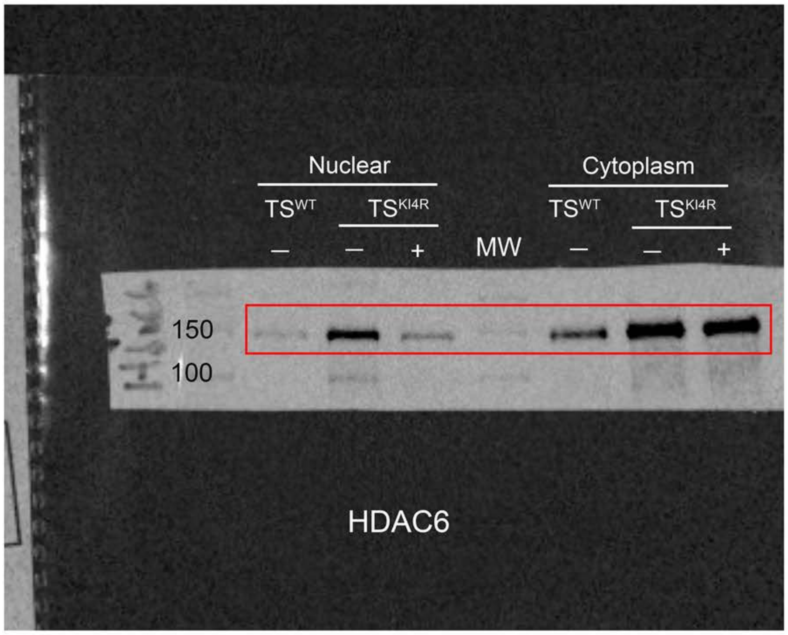
Nuclear
Cytoplasm
TS^{WT}
TS^{KI4R}
TS^{WT}
TS^{KI4R}
– – + MW – – +
150
100
HDAC6

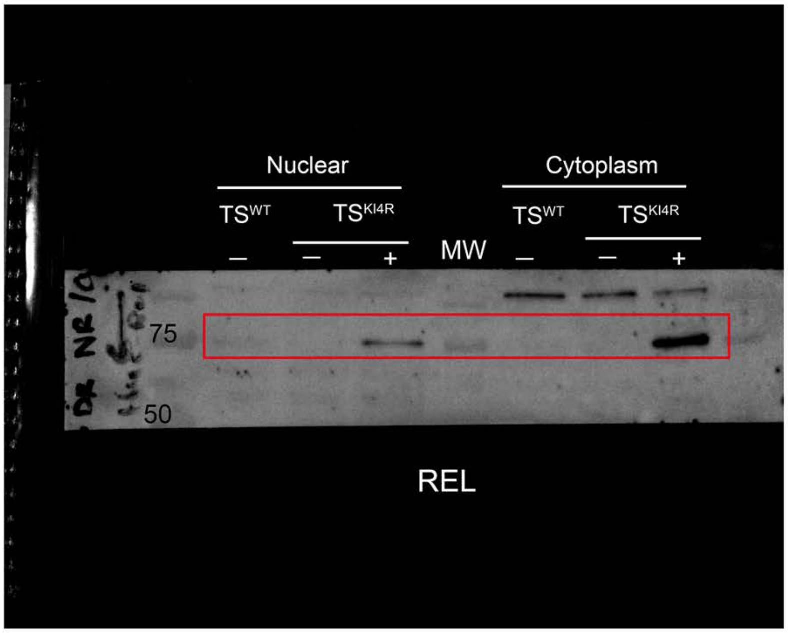
Nuclear
Cytoplasm
TS^{WT}
TS^{KI4R}
TS^{WT}
TS^{KI4R}
– – + MW – – +
75
50
REL

Fig. 7g

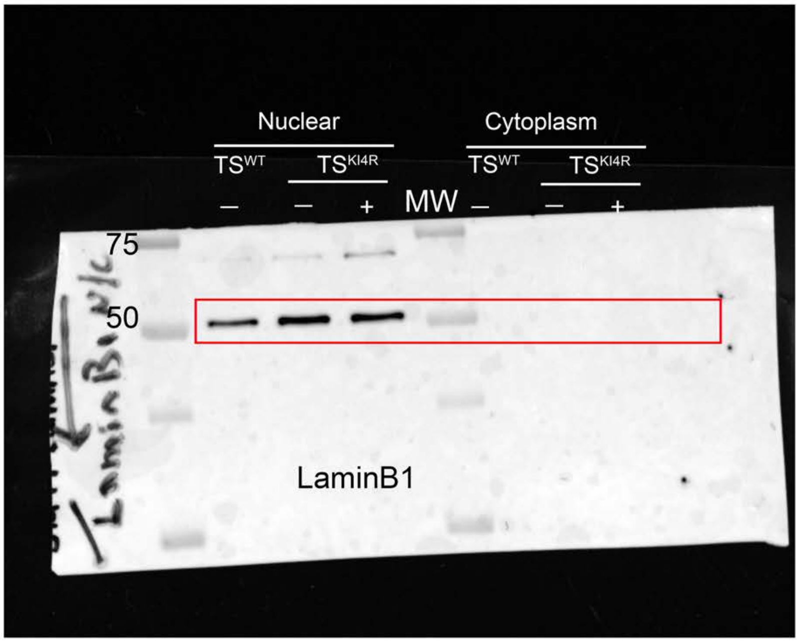
Nuclear
Cytoplasm
TS^WT
TS^KI4R
TS^WT
TS^KI4R
–
–
+
MW
–
–
+
75
50
LaminB1

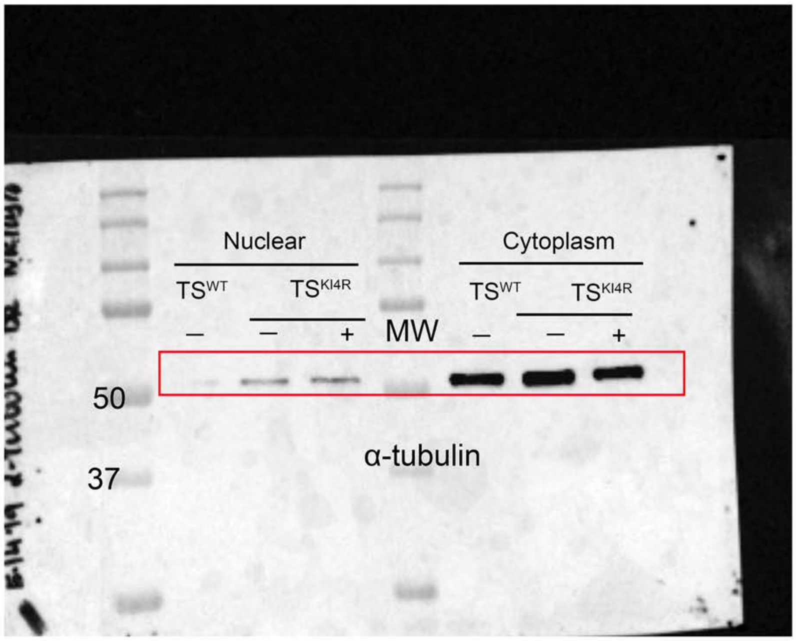
Nuclear
Cytoplasm
TS^WT
TS^KI4R
TS^WT
TS^KI4R
–
–
+
MW
–
–
+
50
37
α-tubulin

Fig. 7h

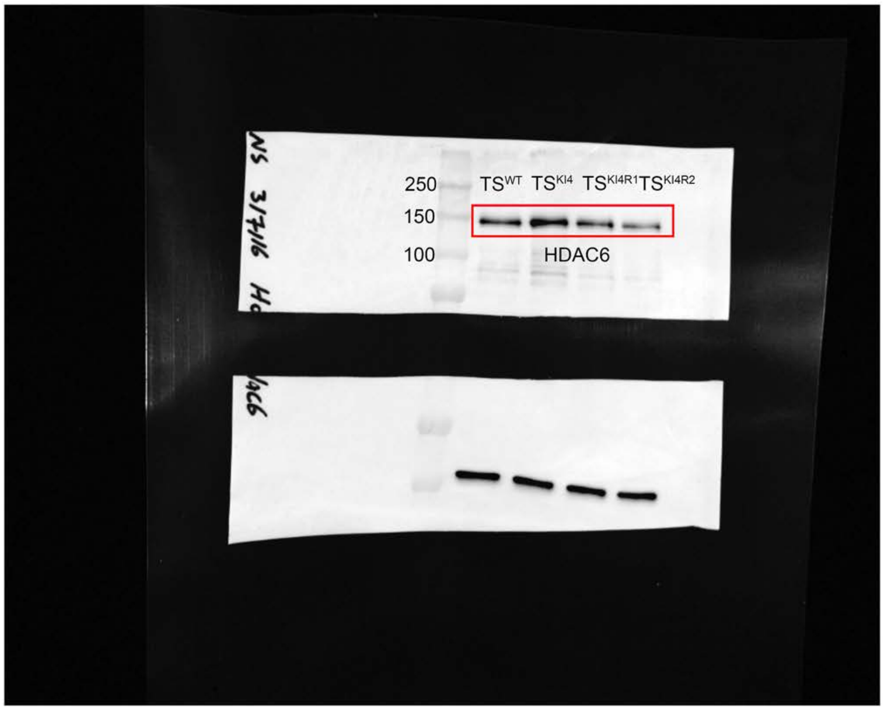
250
150
100
TSWT TSKI4 TSKI4R1 TSKI4R2
HDAC6

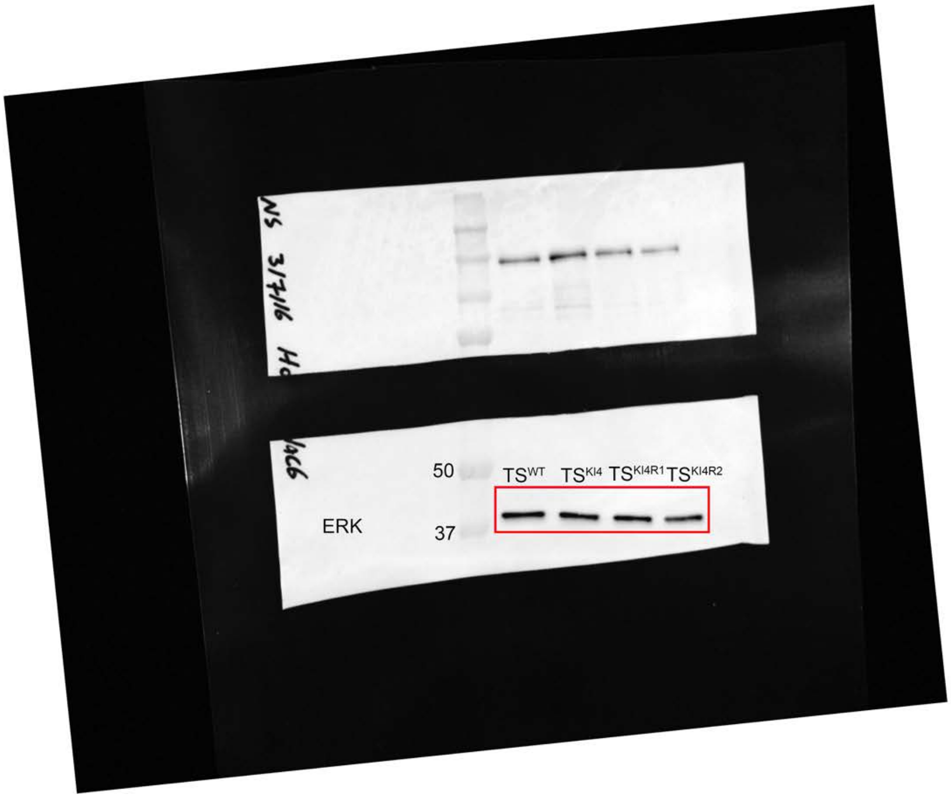
50
37
TSWT TSKI4 TSKI4R1 TSKI4R2
ERK

Fig. 7h

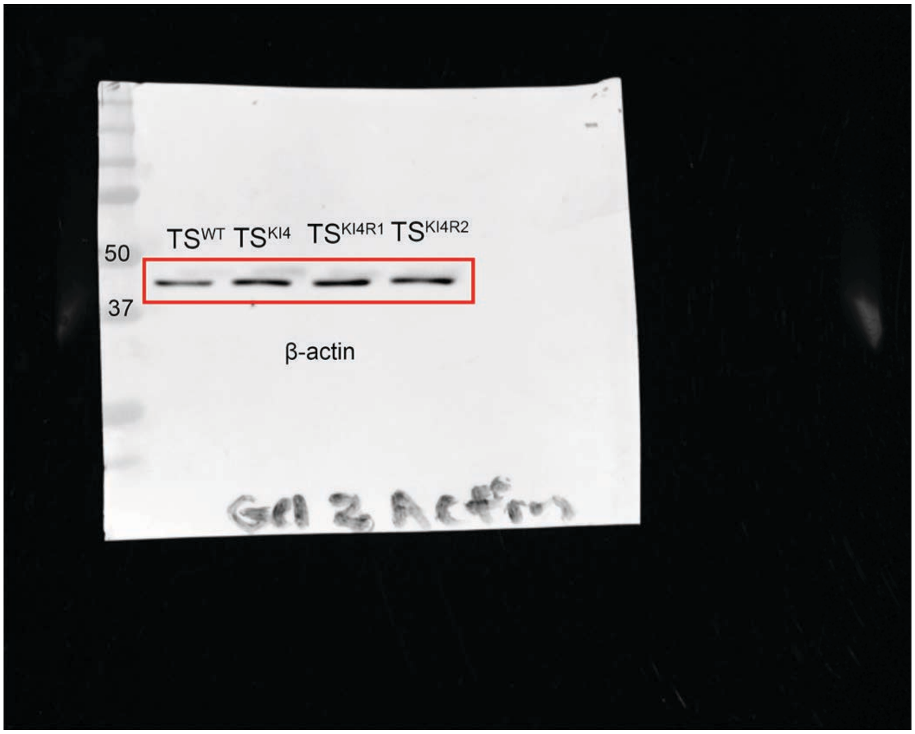

Supplementary Fig. 2b

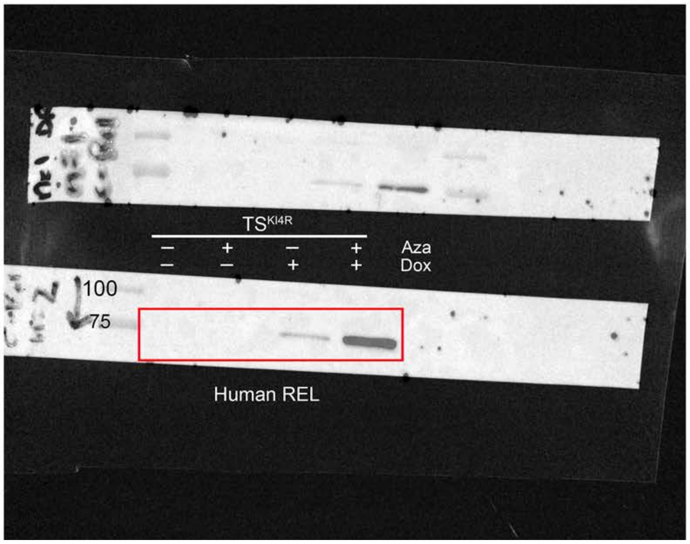

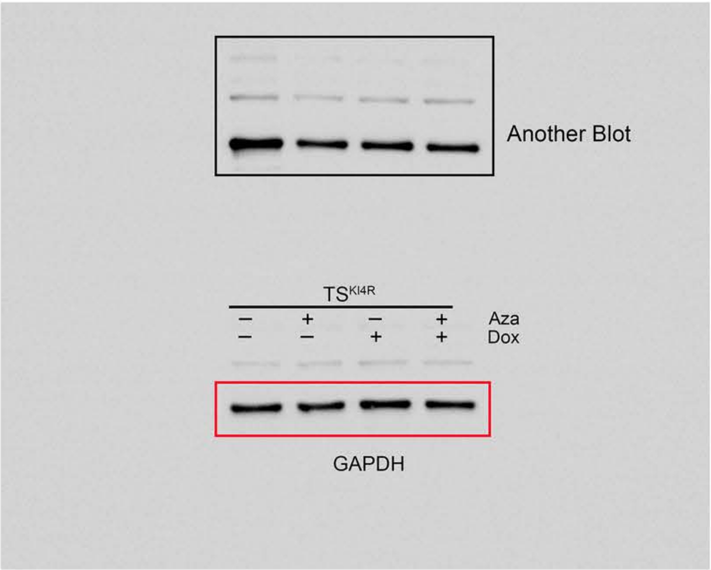

Supplementary Fig. 2d

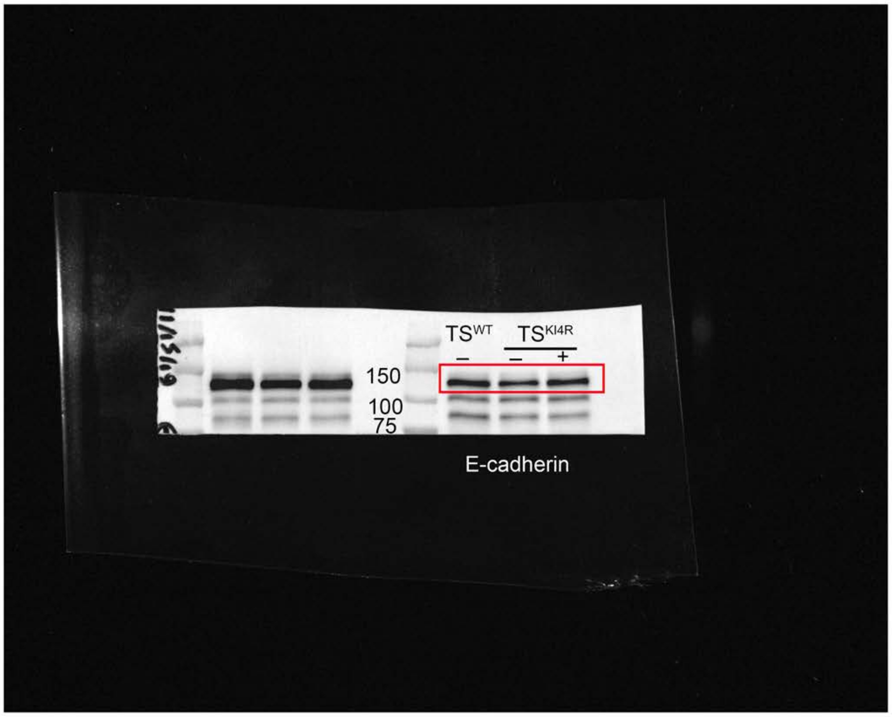

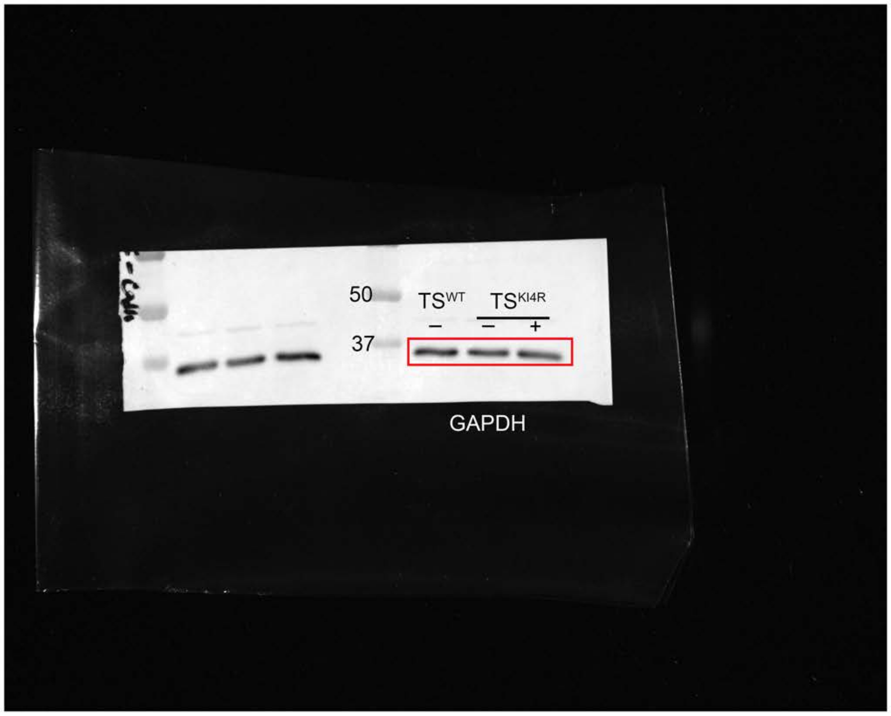

Supplementary Fig. 3a

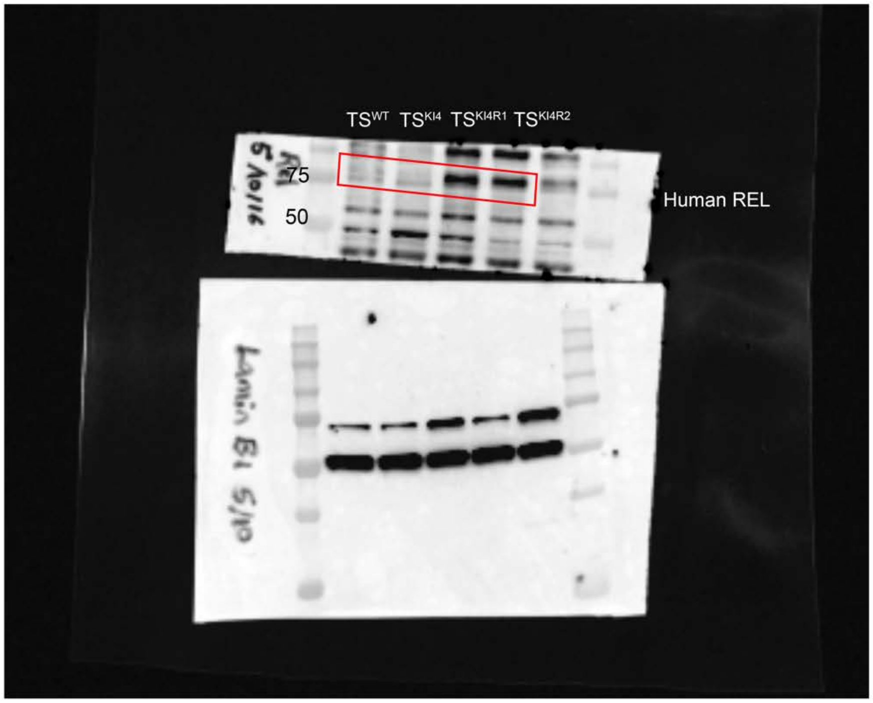

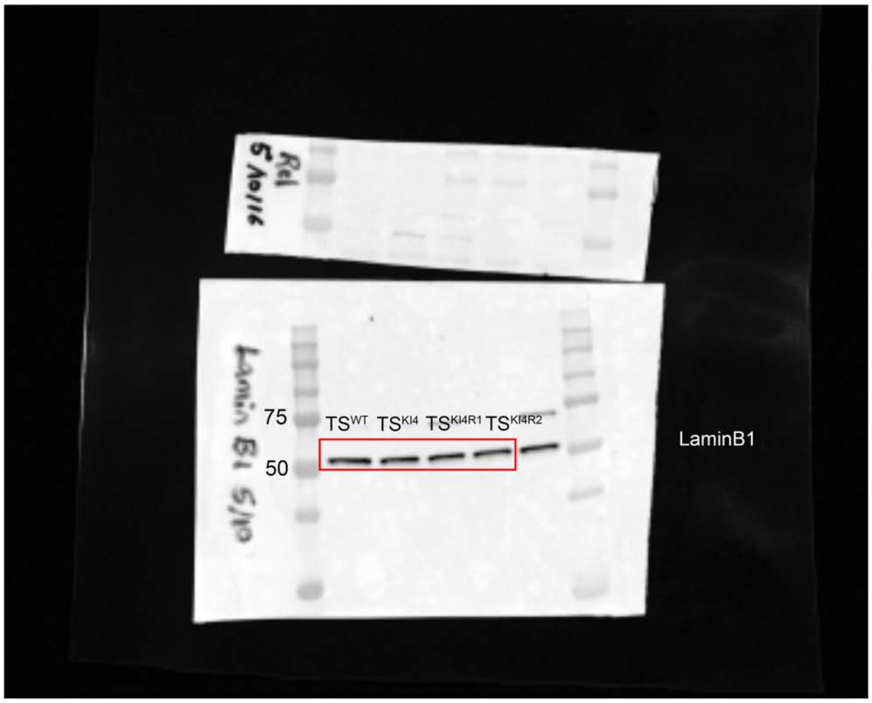

Supplementary Fig. 5d

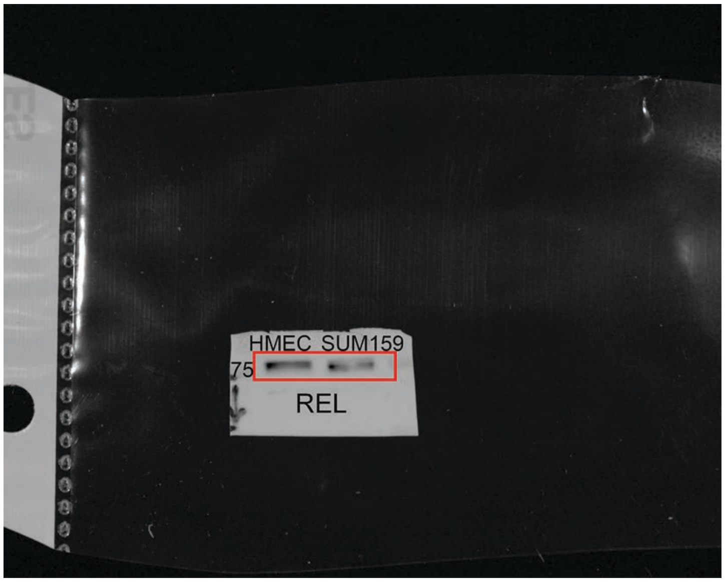

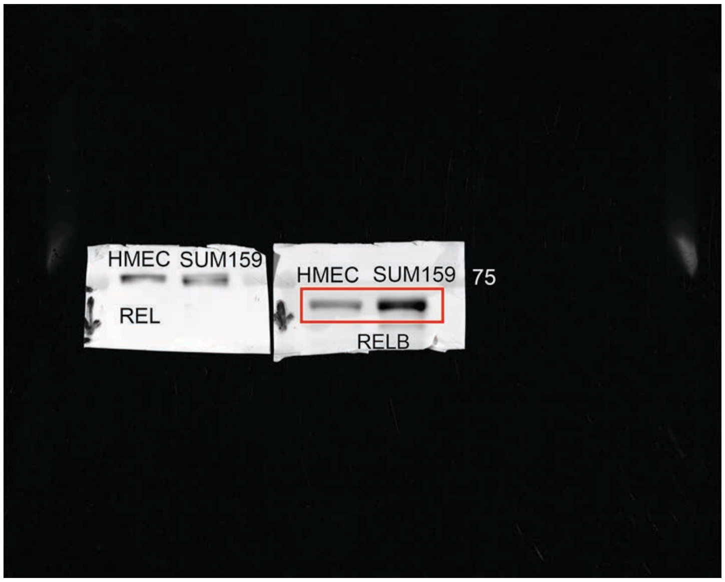

Supplementary Fig. 5d

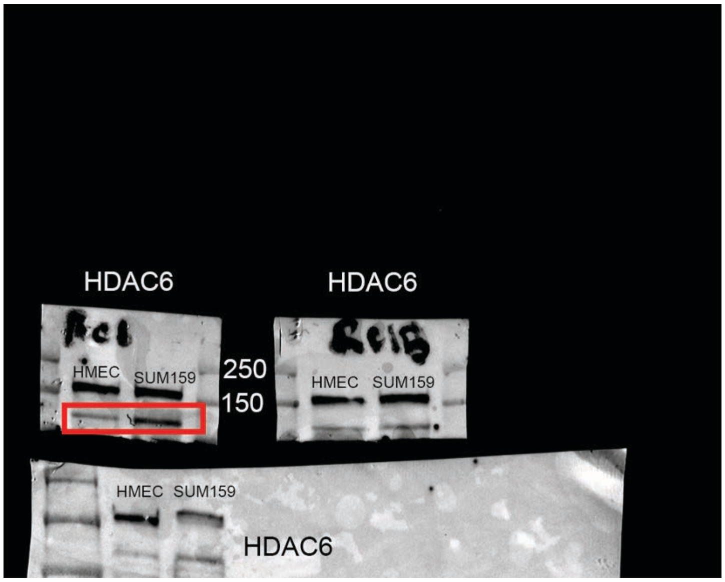

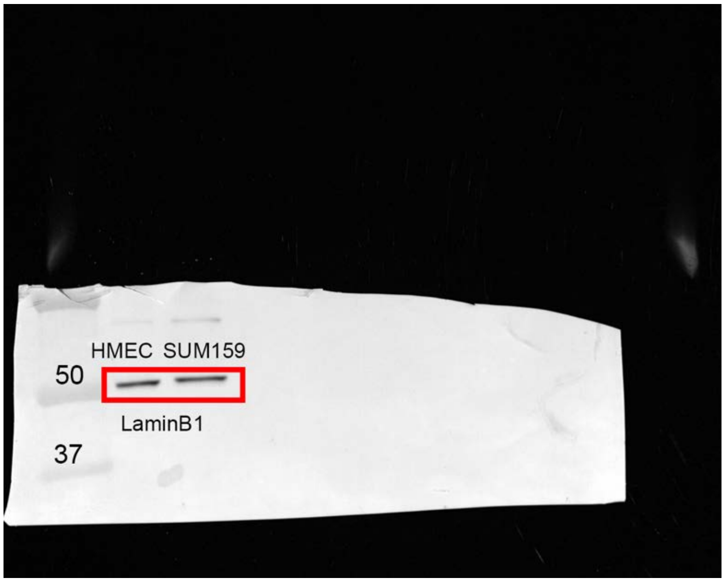

Supplementary Fig. 5d

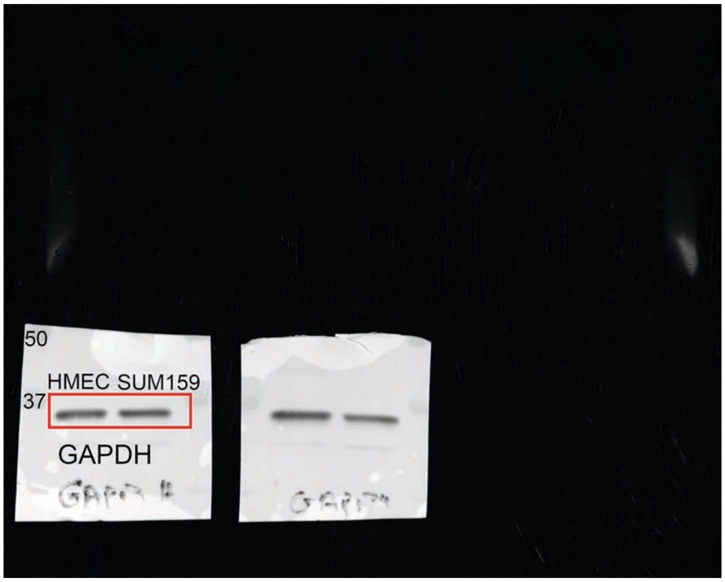

Supplementary Fig. 6e

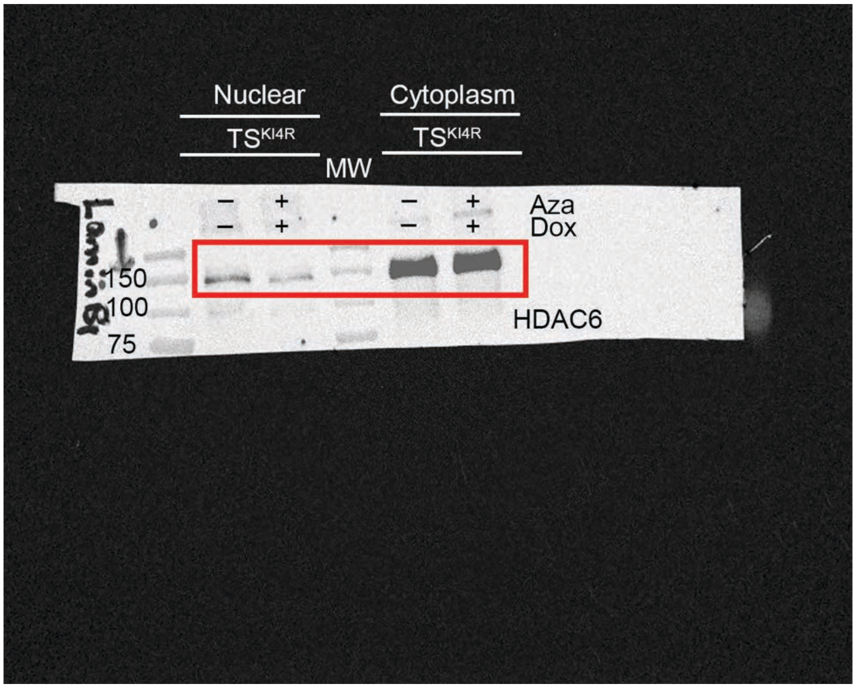

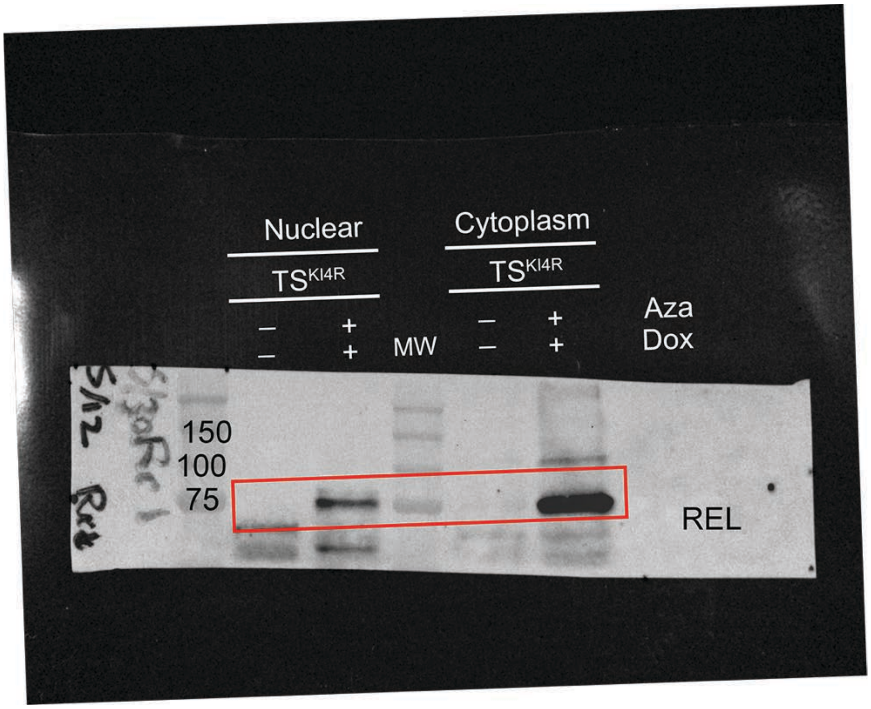

Supplementary Fig. 6e

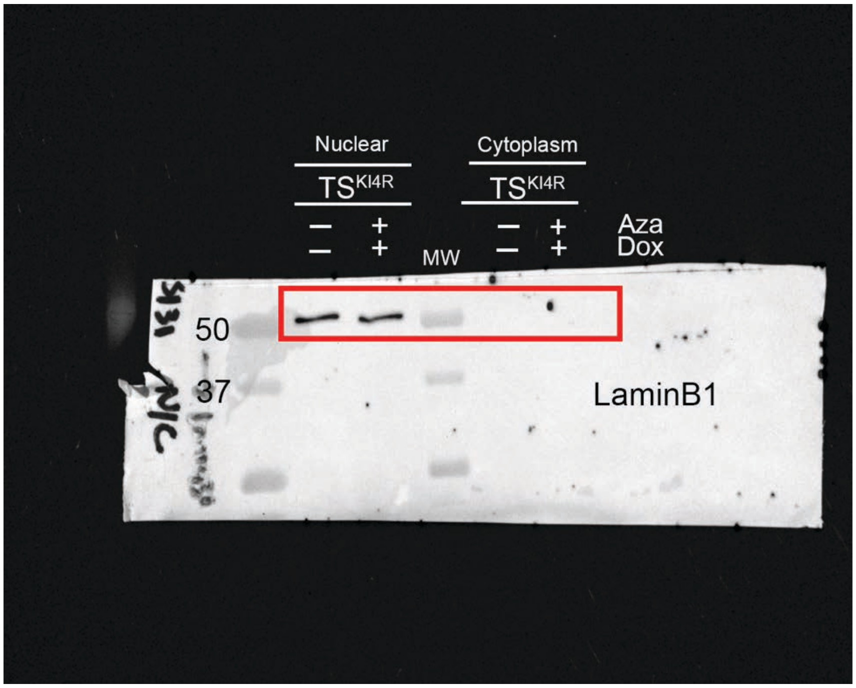

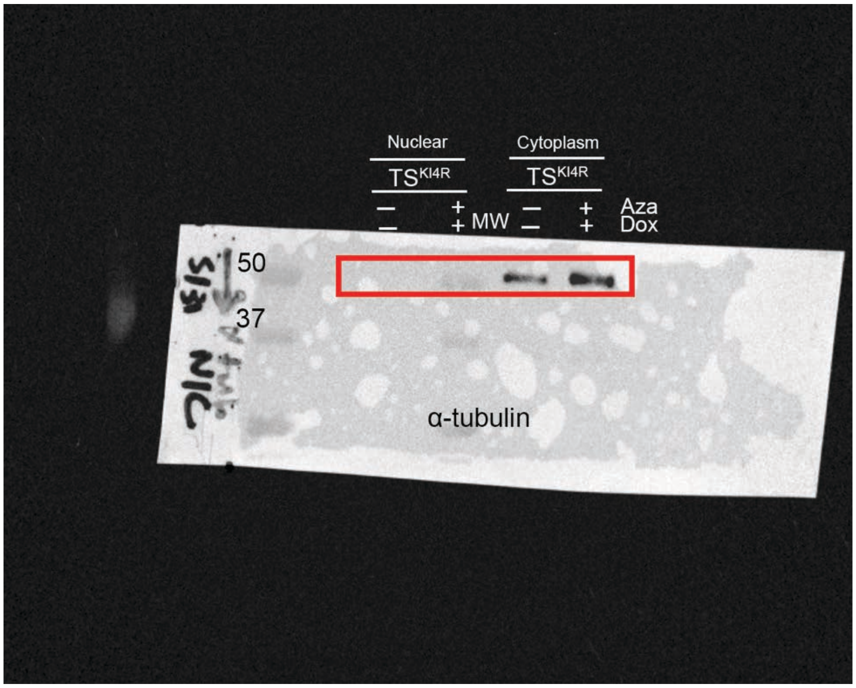

Supplementary Tables

Supplementary Table 1 Representative genes whose expression is either CBP dependent or HDAC6 dependent

Gene name \ RPKM	TS^{WT}	$TS^{WTCBPsh}$	TS^{KI4}	$TS^{KI4H6sh}$
Atf4	146.866	109.348	89.196	92.168
Irx3	56.216	33.050	39.216	24.125
Jdp2	21.460	7.531	2.764	2.630
Itgb2	0.248	0.016	0.022	0.018
Cldn6	122.076	152.681	27.753	139.987
Col4a2	61.2856	66.955	15.413	39.712
Tjp2	46.650	51.626	34.502	57.735
Ocln	0.391	0.930	0.189	1.039

Supplementary Table 2 Antibodies

Antibody	Vendor	Catalog Number	Application Dilution/Concentration
Donkey anti-mouse peroxidase conjugate	Jackson ImmunoResearch Labs	715-035-151	Western blotting 0.26 μg/ ml
Donkey anti-rabbit peroxidase conjugate	Jackson ImmunoResearch Labs	711-035-152	Western blotting 0.26 μg/ml
Goat anti-mouse polyclonal Daylight 488	Thermo Fisher Scientific	35503	Immunofluorescence 2 μg/ml
Mouse monoclonal anti-Actin	Sigma-Aldrich	A4700	Western blotting 1:10,000
Mouse monoclonal anti-E-cadherin	BD Biosciences	610181	Immunofluorescence 0.5 μg/ml
Mouse polyclonal anti-ERK2	Santa Cruz Biotechnology	SC-154	Western blotting 1:10,000
Mouse monoclonal anti-GAPDH	Thermo Fisher Scientific	AM4300	Western blotting 0.1 μg/ml
Mouse monoclonal anti-Tubulin	Sigma-Aldrich	T6793	Western blotting 1:5000
Rabbit monoclonal anti-CBP	Cell Signaling Technology	7425	ChIP 10 μl/ 10 μg chromatin
Rabbit monoclonal anti-CBP	Cell Signaling Technology	7389	Western blotting 1:1000
Rabbit polyclonal anti-c-Rel	Cell Signaling Technology	4727	Western blotting 1:1000
Rabbit monoclonal anti-c-REL	Cell Signaling Technology	12707	Western blotting 1:1000
Rabbit polyclonal anti-c-REL	Santa Cruz Biotechnology	Sc-71	ChIP 1 μg/ 10 μg chromatin
Rabbit polyclonal anti-HDAC6	Bethyl	A301-342A	ChIP 1 μg/ 10 μg chromatin

Rabbit monoclonal anti-HDAC6	Cell Signaling Technology	7612	Western blotting (1:3000)<br>ChIP 5 μl/ 80 μg chromatin
Rabbit monoclonal anti-H2BK5Ac	Active Motif	39123	ChIP<br>1 μl/ 10 μg chromatin
Rabbit monoclonal anti-H3K27Ac	Active Motif	39133	ChIP<br>1 μg/ 10 μg chromatin
Rabbit polyclonal anti-IgG	Abcam	ab171870	ChIP<br>1 μg/ 10 μg chromatin
Rabbit monoclonal anti-Lamin B1	Cell Signaling Technology	13435	Western blotting<br>1:3000
Rabbit polyclonal anti-NF-κB1 (p105/p50)	Cell Signaling Technology	12540	Western blotting<br>1:1000
Rabbit polyclonal anti-NF-κB2 (p100/p52)	Cell Signaling Technology	4882	Western blotting<br>1:1000
Rabbit monoclonal anti-RELA	Cell Signaling Technology	8242	Western blotting<br>1:1000
Rabbit monoclonal anti-RELB	Cell Signaling Technology	4922	Western blotting<br>1:3000

Supplementary Table 3 Primers for qPCR

Target name	Forward primer	Reverse primer
REL<br>Human	GCC TCC GGT GCG TAT AAC C	TGC TGA TCG CCC TTC ACA TT
RELB<br>Human	CAC TCT CGC TCG CCG TTT	CGA AGC CGT TCT CCT TGA TG
2610008E11Rik<br>mouse	GAT TTG AGC CGT GGA ATG GC	GTT CTG CTT CAA CCA TCT GTT CA
Actb<br>mouse	AGC CAT GTA CGT AGC CAT CC	CTC TCA GCT GTG GTG GTG AA
Cdh1<br>mouse	CAG GTC TCC TCA TGG CTT TGC	CTT CCG AAA AGA AGG CTG TCC
Cdh2<br>mouse	AGC GCA GTC TTA CCG AAG G	TCG CTG CTT TCA TAC TGA ACT TT
Cdx2<br>mouse	AAG CCA AGT GAA AAC CAG GAC A	GGC AGC CAG CTC ACT TTT C
Cldn6<br>mouse	ATG GCC TCT ACT GGT CTG CAA	GCC AAC AGT GAG TCA TAC ACC TT
Crebl2<br>mouse	TGG ATG ACA GCA AGG TGG TT	GAT TTT GGC TGG CTT CCG TC
Crebbp<br>mouse	GGC TTC TCC GCG AAT GAC AA	GTT TGG ACG CAG CAT CTG GA
Ets1<br>mouse	CTC TCC AGA CAG ACA CCT TGC	CGG CCC ACT TCC ACG ACT
Gapdh<br>mouse	AGG TCG GTG TGA ACG GAT TTG	TGT AGA CCA TGT AGT TGA GGT CA
Gli1<br>mouse	ATC ACC TGT TGG GGA TGC TG	AAT CGA ACT CCT GGC TGC AA
Hivep2<br>mouse	GGC TCG GTG AAG TCT GTG AG	CTG GCT GGC TGT TGA GAG AA
HoxA1<br>mouse	CAG GAA GCA GAC CCA CCA AG	ACC CAC GTA GCC GTA CTC T
Id2<br>mouse	ATG AAA GCC TTC AGT CCG GTG	AGC AGA CTC ATC GGG TCG
Krt8<br>mouse	CGG CTA CTC AGG AGG ACT GA	TGA AAG TGT TGG ATC CCC CG
Krt18<br>mouse	GTG GAT GCC CCC AAA TCT CA	CCT CAA TCT GCT GAG ACC AGT
Nf□b1<br>mouse	ATG GCA GAC GAT GAT CC TAC	TGT TGA CAG TGG TATT TCT GGT G
Nf□b2<br>mouse	GGC CGG AAG ACC TAT CC ACT	CTA CAG ACA CAG CGC ACA CT

Pou6f1 mouse	CTC CAA GAT CAG TGC AGC CTC C	CGT GAG GAT CTG ACT GCT GA
Rel mouse	ACC TCA ATG TGG TGA GGC TG	TT GGG GCA CGG TTG TCA TAA
RelA mouse	AGG CTT CTG GGC CTT AT TG	TGC TTC TCT CGC CAG GAA TAC
RelB mouse	CTC CAT TCC GAA GCC AAC CT	GGC TCA AAG AGA ACC GGA GT
Rfx3 mouse	CCT CCA GCT GTC TCA GAG ATT T	GTC TGA ACC CGT CTC TGA AGT
Rps11 mouse	CGC GTG GTG AAT AAG GAA GC	GTA AGC ACG CTC CGT CTG AA
Runx1 mouse	GC AGG CAA CGA TGA AAA CTA CT	GCA ACT TGT GGC GGA TTT GTA
Snai2 mouse	TGG TCA AGA AAC ATT TCA ACG CC	GGT GAG GAT CTC TGG TTT TGG TA
Tlx1 mouse	GCT TGC CTA CAG TAC CCT CTG	TAG GGG TGA CCT GTG AAC CT
Twist1 mouse	GGA CAA GCT GAG CAA GAT TCA	CGG AGA AGG CGT AGC TGA G
Vim mouse	TCC ACA CGC ACC TAC AGT CT	CCG AGG ACC GGG TCA CAT A
Zeb1 mouse	ACC GCC GTC ATT TAT CCT GAG	CAT CTG GTG TTC CGT TTT CAT CA
Zfp672 mouse	TAT CCC AGG CTG CAG GTA CA	ACC TTT GGG GAG CTT CGT TT
Zfp810 mouse	CGA CTC CGC CAT GAT GTA GA	GTC ATG CAG TGC CCA TTT CC

Supplementary Table 4 ChIP-PCR primers for mouse *Rel* promoter and distal regulatory regions where numbers indicate the distance from mouse *Rel* TSS

Target name	Forward primer	Reverse primer
-700	GAG ATT GGC CGA GAT ACC CA	GCA AAC AAG ATT CCG CCT GA
+140699	CAG AGG GAG GCT GTT AGT CCT	CTT TCA GAA TCA GAG CCA ACC G
+105579	CTG CGG CAT CTT GGG AGA A	TCT TCG ACA CCC AGT CCC TA
+101930	TCC CGC ACC TTA CAA ACA CC	ACA GGA CTC AAA GCT AGT GAC A
+83091	GTT TTC TCC TTT TCC CTC CCT	TAG GAC ATA CCA GCT TTT ACG G
+71150	CCC TCT GGC TTT GCA CCA TA	CCC CTG TCA CAC ATG CTG AA
+46498	GAA CTC GGG CCA TTC ACT CT	GGC AGA AGG ATA CGT TCC AG
+40681	TTG TGT GGG CCG GAA TAG AT	CAC TGC AAC TCA CCC TCA CT
+35817	GTG GGA TTA CTT CAA GGA TGG GT	TAC ATA TCT TTG ATG GGT CTG CTA A
+33455	GTT GGT CTG AGC TGA GCA GAT A	GAG GTT TGT GTG GTG CTA GAG
+29050	GAT GGG CCA CTG AGG GTA TG	CCA GGG CAA GCT GAA CCT TA
-9753	TCA GGA ATA AAA GAA GGC ACT CTA	TGA ATT TCT TAT CTA GGC CCA CAC T
-37106	AGT CTG AGC TGT GCT TTA CGG	TCT GTG CTG GAG TTC CCC TAC
-120247	TCT CCC TGG TCT GTG GGA TT	GCA GAA ACA AGG GGA AAT TGA CA

-297506	AGT TTC CAC CGA GAC AGA CC	CAG TGA GAG CAT CCC GTA CA

Supplementary Table 5 ChIP-PCR primers for Human *REL* promoter

Target name	Forward primer	Reverse primer
Human *REL* promoter	GCT CAA GGT TCC TAA TCT GAC G	TCT TTC CCC TTA TTT CCA GCG A
