# Supplementary material for: Coordinated regulation of Rel expression by MAP3K4, CBP, and HDAC6 controls phenotypic switching
Source: Commun Biol. 2020 Aug 28;3:475. doi: 10.1038/s42003-020-01200-z (PMC7455715; doi:10.1038/s42003-020-01200-z)
Supplement: Supplementary file 2 — Description of Additional Supplementary Files [file 42003_2020_1200_MOESM2_ESM.docx]

**Descriptions of Additional Supplementary Files**

**Supplementary Data 1** related to Fig. 1: Gene names of the 183 co-regulated genes. Excel sheet lists the gene names of the 183 genes whose expression is co-regulated by MAP3K4, CBP, HDAC6, and H2BK5Ac. These 183 genes were identified as described in Fig. 1 and Supplementary Fig. 1.

**Supplementary Data 2** related to Fig. 7: Expression of inflammatory cytokines and receptors in TS cells. Excel sheet lists the gene names and RPKM values derived from RNAseq for genes encoding cytokines and their receptors. RNAseq was performed on wild-type TS cells (TSWT), wild-type TS cells expressing Crebbp shRNA (TSWTCBPsh), kinase inactive MAP3K4 TS cells (TSKI4), and TSKI4 cells expressing Hdac6 shRNA (TSKI4H6sh).

**Supplementary Data 3**: Raw data for all graphs and figures in the main manuscript.

**Supplementary Movie 1** related to Fig. 3: Live cell imaging of TSWT cells shows stationary, growing cells. Wild-type TS cells were cultured on 6-well tissue culture plastic dishes. Cells were placed in a humidified Lionheart FX live cell imager heated to 37C and having 7% CO2. The 3 x 3 images were captured with a 20X objective every five minutes for 28 hours. These images were stitched together to create the video.

**Supplementary Movie 2** related to Fig. 3: Live cell imaging of TSKI4R-Dox cells shows actively migrating cells. TSKI4 cells were transduced with a lentiviral construct for the Doxinducible expression of human REL (TSKI4R cells). TSKI4R-Dox cells were cultured on 6-well tissue culture plastic dishes in the absence of Dox. Cell were placed in a humidified Lionheart FX live cell imager heated to 37C and having 7% CO2. The 3 x 3 images were captured with a 20X objective every five minutes for 28 hours. These images were stitched together to create the video.

**Supplementary Movie 3** related to Fig. 3: Live cell imaging of TSKI4R+Dox cells shows REL induction reduces cell motility. TSKI4 cells transduced with a lentiviral construct for the Doxinducible expression of human REL (TSKI4R cells) were cultured in the presence of Dox to induce REL expression. Cells were cultured on 6-well tissue culture plastic dishes. Cells were placed in a humidified Lionheart FX live cell imager heated to 37C and having 7% CO2. The 3 x 3 images were captured with a 20X objective every five minutes for 28 hours. These images were stitched together to create the video

**Supplementary Movie 4** related to Fig. 4: Live cell imaging of TSKI4RB cells shows RELB expression increases cell motility. TSKI4 cells were transduced with a lentiviral construct expressing human RELB. TSKI4RB cells were cultured on 6-well tissue culture plastic dishes. Cells were placed in a humidified Lionheart FX live cell imager heated to 37C and 7% CO2. The 3 x 3 images were captured with a 20X objective every five minutes for 28 hours. These images were stitched together to create the video.
