# Supplementary material for: Coordinated regulation of Rel expression by MAP3K4, CBP, and HDAC6 controls phenotypic switching
Source: Commun Biol. 2020 Aug 28;3:475. doi: 10.1038/s42003-020-01200-z (PMC7455715; doi:10.1038/s42003-020-01200-z)
Supplement: Supplementary file 10 — Reporting Summary [file 42003_2020_1200_MOESM10_ESM.pdf]

nature research

Corresponding author(s): Amy N. Abell

Last updated by author(s): Jul 6, 2020

Reporting Summary

Nature Research wishes to improve the reproducibility of the work that we publish. This form provides structure for consistency and transparency in reporting. For further information on Nature Research policies, see Authors & Referees and the Editorial Policy Checklist.

Statistics

For all statistical analyses, confirm that the following items are present in the figure legend, table legend, main text, or Methods section.

n/a	Confirmed	
☐	☒	The exact sample size (n) for each experimental group/condition, given as a discrete number and unit of measurement
☐	☒	A statement on whether measurements were taken from distinct samples or whether the same sample was measured repeatedly
☐	☒	The statistical test(s) used AND whether they are one- or two-sided<br>*Only common tests should be described solely by name; describe more complex techniques in the Methods section.*
☒	☐	A description of all covariates tested
☒	☐	A description of any assumptions or corrections, such as tests of normality and adjustment for multiple comparisons
☐	☒	A full description of the statistical parameters including central tendency (e.g. means) or other basic estimates (e.g. regression coefficient) AND variation (e.g. standard deviation) or associated estimates of uncertainty (e.g. confidence intervals)
☐	☒	For null hypothesis testing, the test statistic (e.g. F, t, r) with confidence intervals, effect sizes, degrees of freedom and P value noted<br>*Give P values as exact values whenever suitable.*
☒	☐	For Bayesian analysis, information on the choice of priors and Markov chain Monte Carlo settings
☒	☐	For hierarchical and complex designs, identification of the appropriate level for tests and full reporting of outcomes
☒	☐	Estimates of effect sizes (e.g. Cohen's d, Pearson's r), indicating how they were calculated

Our web collection on statistics for biologists contains articles on many of the points above.

Software and code

Policy information about availability of computer code

Data collection	Bio-Rad ChemiDoc Version 2.3.0.07, commercially available software from Bio-Rad to use and run the Bio-Rad ChemiDoc Touch Imager.
Data analysis	Bio-Rad Image Lab 5.2.1, commercially available software from Bio-Rad used to analyze images of gels and blots acquired using the Bio-Rad ChemiDoc Touch Imager.<br>Bio-Rad CFX Maestro software 4.0.2325.2, commercially available software from Bio-Rad used to analyze qPCR data acquired with a Bio-Rad CFX96 Touch qPCR machine.

For manuscripts utilizing custom algorithms or software that are central to the research but not yet described in published literature, software must be made available to editors/reviewers. We strongly encourage code deposition in a community repository (e.g. GitHub). See the Nature Research guidelines for submitting code & software for further information.

Data

Policy information about availability of data

All manuscripts must include a data availability statement. This statement should provide the following information, where applicable:

- Accession codes, unique identifiers, or web links for publicly available datasets
- A list of figures that have associated raw data
- A description of any restrictions on data availability

The sequencing and microarray data were deposited in the NCBI Gene Expression Omnibus (GEO). The accession number for all new sequencing data reported in this paper is GEO: GSE148496. The accession number for microarray data reported in this paper is GEO: GSE148250. The accession number for previously reported promoter capture Hi-C data deposited in ArrayExpress is E-MTAB-6585. The raw source data for the main figures are included in the Supplementary Source Data file provided in Supplementary Information. Any other data not included in the paper or supplementary materials is available from the authors upon reasonable request. The corresponding author, Dr. Amy Abell, will provide the requested data.

Field-specific reporting

Please select the one below that is the best fit for your research. If you are not sure, read the appropriate sections before making your selection.

☒ Life sciences ☐ Behavioural & social sciences ☐ Ecological, evolutionary & environmental sciences

For a reference copy of the document with all sections, see nature.com/documents/nr-reporting-summary-flat.pdf

Life sciences study design

All studies must disclose on these points even when the disclosure is negative.

Sample size	Sample size calculations were not performed. Sample sizes ranged between two and five independent experiments. Sample size was chosen based on the need for statistical analyses.
Data exclusions	No data was excluded from the analyses.
Replication	Between two and five independent experiments were performed as necessary to verify the reproducibility of the data. Information regarding replication of experiments is provided in each figure legend.
Randomization	Experiments were not randomized, because samples for each independent experiment were isolated together, and the reagents are independent of each other.
Blinding	Experiments were not blinded.

Reporting for specific materials, systems and methods

We require information from authors about some types of materials, experimental systems and methods used in many studies. Here, indicate whether each material, system or method listed is relevant to your study. If you are not sure if a list item applies to your research, read the appropriate section before selecting a response.

Materials & experimental systems

n/a	Involved in the study	
☐	☒	Antibodies
☐	☒	Eukaryotic cell lines
☒	☐	Palaeontology
☒	☐	Animals and other organisms
☒	☐	Human research participants
☒	☐	Clinical data

Methods

n/a	Involved in the study	
☒	☐	ChIP-seq
☒	☐	Flow cytometry
☒	☐	MRI-based neuroimaging

Antibodies

Antibodies used

Donkey anti-mouse peroxidase conjugate Jackson ImmunoResearch Labs Cat#715-035-151 Lot#130080 Clone#NA

Donkey anti-rabbit peroxidase conjugate Jackson ImmunoResearch Labs Cat#711-035-152 Lot#130173 Clone#NA

Goat anti-mouse polyclonal Daylight 488 Thermo Fisher Scientific Cat#35503 Lot#OE188374 Clone#NA

Mouse monoclonal anti-Actin Sigma-Aldrich Cat#A4700 lot#035M4757V Clone#AC-40

Mouse polyclonal anti-ERK2 Santa Cruz Biotechnology Cat #SC-154 Lot#F0713

Mouse monoclonal anti-E-cadherin BD Biosciences Cat#610181 Lot#7187865 Clone#36

Mouse monoclonal anti-GAPDH Thermo Fisher Scientific Cat#AM4300 Lot#00689657 Clone#6C5

Mouse monoclonal anti-Tubulin Sigma-Aldrich Cat#T6793 Lot#115M4827V Clone#DM1A

Rabbit monoclonal anti-CBP Cell Signaling Technology Cat#7425 Lot#1 Clone#D9B6

Rabbit monoclonal anti-CBP Cell Signaling Technology Cat#7389 Lot#1 Clone#D6C5

Rabbit polyclonal anti-c-Rel Cell Signaling Technology Cat#4727 Lot#4 Clone#NA

Rabbit monoclonal anti-c-REL Cell Signaling Technology Cat#12707 Lot#11 Clone#D4X6M

Rabbit polyclonal anti-c-REL Santa Cruz Biotechnology Cat#Sc-71 Lot#A2916 Clone#C

Rabbit polyclonal anti-HDAC6 Bethyl Cat#A301-342A LotA301-342A-2 Clone#NA

Rabbit monoclonal anti-HDAC6 Cell Signaling Technology Cat#7612 Lot#2 Clone#D21B10

Rabbit monoclonal anti-H2BK5Ac Active Motif Cat#39123 Lot#01008001 Clone#NA

Rabbit monoclonal anti-H3K27Ac Active Motif Cat#39133 Lot#131814008 Clone#NA

Rabbit polyclonal anti-IgG Abcam Cat#ab171870 Lot#GR3183459-1 Clone#NA

Rabbit monoclonal anti-Lamin B1 Cell Signaling Technology Cat#13435 Lot#2 Clone#D9V6H

Rabbit polyclonal anti-NF-κB1 (p105/p50) Cell Signaling Technology Cat#12540 Lot#2 Clone#D7H5M

Rabbit polyclonal anti-NF-κB2 (p100/p52) Cell Signaling Technology Cat#4882 Lot#4 Clone#NA

Rabbit monoclonal anti-RELA Cell Signaling Technology Cat#8242 Lot#9 Clone#D14E12

Rabbit monoclonal anti-RELB Cell Signaling Technology Cat#4922 Lot#3 Clone#C1E4

Validation

Validation of the antibodies was based on the manufacturer's information. Rabbit polyclonal anti-HDAC6 was validated for ChIP-PCR by comparing it to isotype control IgG antibody.
Antibodies were validated by the manufacturer.

Eukaryotic cell lines

Policy information about cell lines

Cell line source(s)

Trophoblast stem cells used in this study were isolated and described in a previous publication from a wild-type conceptus and from a conceptus homozygous for a mutation in MAP3K4 (K1361R). Reference: Abell, A.N., Granger, D.A., Johnson, N.L., Vincent-Jordan, N., Dibble, C.F., and Johnson, G.L., (2009). Trophoblast stem cell maintenance by fibroblast growth factor 4 requires MEKK4 activation of jun N-terminal kinase. Mol Cell Biol. 29, 2748-2761. PMCID: PMC2682043. Immortalized human mammary epithelial cells (HMECs) were created by Christopher Counter at Duke University. Reference: Troester, M. A., Hoadley, K. A., Sorlie, T., Herbert, B. S., Borresen-Dale, A. L., Lonning, P. E., Shay, J. W., Kaufmann, W. K., and Perou, C. M. (2004). Cell-type-specific responses to chemotherapeutics in breast cancer. Cancer Research 64, 4218-26. HMECs and human SUM159PT breast cancer cells were obtained from Dr. Gary Johnson as published in Reference: Duncan, J.S., Whittle, M.C., Nakamura, K., Abell, A.N., Midland, A.A., Zawistowski, J.S., Johnson, N.L., Granger, D.A., Jordan, N.V., Darr, D.B., Usary, J., Kuan, P.F., Smalley, D.M., Major, B., He, X., Hoadley, K.A., Zhou, B., Sharpless, N.E., Perou, C.M., Kim, W.Y., Gomez, S.M., Chen, X., Jin, J., Frye, S.V., Earp, H.S., Graves, L.M., and Johnson, G.L., (2012). Dynamic reprogramming of the kinome in response to targeted MEK inhibition in triple negative breast cancer. Cell 149, 307-321. PMCID: PMC3328787.

Authentication

TS cell lines were tested for the expression of stemenss markers for TS cells and the ability to differentiate into mature trophoblasts.

Mycoplasma contamination

All cell lines tested negative for mycoplasma.

Commonly misidentified lines
(See ICLAC register)

None
